# Person-centred quality indicators for Australian aged care assessment services: a mixed methods study

**DOI:** 10.1186/s40900-024-00606-x

**Published:** 2024-08-14

**Authors:** Sandra Smith, Catherine Travers, Melinda Martin-Khan, Ivy Webb, Elizabeth Miller, Jane Thompson, Natasha Roberts

**Affiliations:** 1 The University of Queensland, Centre for Health Services Research, Brisbane, Australia; 2 School of Health and Rehabilitation Sciences, The University of Queensland, Brisbane, Australia; 3 Centre for Clinical Research, The University of Queensland, Brisbane, Australia; 4 STARS Education and Research Alliance, Surgical Treatment and Rehabilitation Service (STARS), The University of Queensland and Metro North Health, Brisbane, Australia; 5 College of Medicine and Health, University of Exeter, Devon, England, UK; 6 Public Contributors, Brisbane, Australia

**Keywords:** Older people, Aged care assessment services, Person-centred quality indicators, Quality measures, Policy

## Abstract

**Background:**

Aged Care Assessment Teams are the assessment component of the Australian aged care system. Their purpose is to undertake needs-based assessments to determine an older person’s eligibility for, and access to Commonwealth-funded aged care services. There are no measures that tell us if the aged care assessment service is of high quality from the perspective of the person being assessed. Quality measures have been developed and introduced in Australian residential aged care facilities. These however, have not considered the perspectives of those living in this setting. Quality measures for home care services have also been recommended.

This research aims to address the gap in person-centred quality measures by asking current and future service users of aged care assessment services to vote on the importance of 24 person-centred quality indicators (PC-QIs), that were developed in a previous study using a modified Delphi method approach supported by engagement with a consumer led Advisory Board.

**Methods:**

This mixed methods study used the RAND/UCLA Appropriateness Method to reach consensus on a final set of PC-QIs. Twenty-five community-dwelling older people in Brisbane, Australia, voted on the importance of 24 PC-QIs using a five-point Likert scale. A consensus statement for PC-QI elimination was determined prior to participants voting. Voting was undertaken with participants individually either face-to-face or via telephone, in their homes. To capture any narrative provided by participants regarding each PC-QI, participant voting sessions were audio-recorded and subsequently transcribed verbatim.

Quantitative data from participant votes for each PC-QI were calculated and statistically described by median, interquartile range, consensus met, percentile, percentile rank, rank order, median and standard deviation. PC-QIs were then assessed against the consensus statement for elimination and rank ordered according to importance to participants. Content analysis of qualitative data from audio transcriptions was conducted to determine the presence of certain words supporting participant votes for each PC-QI.

**Results:**

No PC-QIs were eliminated during voting. Variation existed among participants’ ratings of importance for each PC-QI. Final quality domains, their respective title, quality indicator descriptor and supporting qualitative data are presented. Five PC-QIs had a median of five, no votes recorded below four, an interquartile range of zero, and a rank order score of one, two and four, out of a possible ten, indicating they were of highest importance to participants.

**Conclusion:**

Participants reached consensus on 24 evidence-based PC-QIs that represent measures of quality of aged care assessment services from the perspectives of current and future service users.

**Supplementary Information:**

The online version contains supplementary material available at 10.1186/s40900-024-00606-x.

## Abstract (plain language summary)

In Australia, people 65 years or over, and Aboriginal and Torres Strait Islander Peoples 50 years or over, can apply to access aged care services funded by the Australian government (Australian Government, Department of Health and Aged Care. 2021-2022 Report on the Operation of the Aged Care Act 1997, 2022). Services the government funds include supporting older people to live at home, residential aged care if the person can’t live at home, and short-term rehabilitation (Australian Government, Department of Health and Aged Care. 2021-2022 Report on the Operation of the Aged Care Act 1997, 2022). To access these services, a person needs to fill out an application form and undergo an aged care assessment. Another person can help complete the application. The purpose of the aged care assessment is to assess the person’s needs to determine what aged care services they are eligible to access. There are no measures that tell us if the aged care assessment service is of high quality from the perspective of the person being assessed. Twenty-four measures of quality were developed in a previous study with support from a consumer Advisory Board (Smith S, Travers C, Roberts N, Martin‐Khan M. Health Expect, 2024). This study asked 25 older people living in Brisbane, the importance of the 24 measures, to decide if any should be eliminated. People were asked to vote using a number scale where number one meant it ‘wasn’t important’, and five meant it was ‘extremely important.’ A consensus statement was agreed to decide if a measure would be eliminated. All measures were voted as being important with no measure eliminated. Quality Measures voted as being important included receiving assessments from knowledgeable health care staff, who treated them with dignity and respect, adopted a person-centred approach, established a collaborative relationship, and communicated clearly.

**Supplementary Information:**

The online version contains supplementary material available at 10.1186/s40900-024-00606-x.

## Introduction

In June 2022, older people (people aged 65 years or greater, 50 years or greater for Indigenous Australians, referred to as ‘older people’ or ‘older Australians’ in this paper) comprised approximately 16.5% (4.4 million) of the total Australian population [1]. By 2032 it is projected that this group will increase to 18.3% (5.6 million) [1]. During 2021–22, Aged Care Assessment Teams (ACATs) across Australia conducted 200,562 assessments to determine an older person’s eligibility for government-funded aged care services [2]. The Australian aged care system funds residential aged care, transition care, home care, short term restorative care and home support services to eligible older Australians [2]. To be eligible, a person must apply for care in accordance with the *Aged Care Act 1997* [3]*,* and have their care needs assessed by an aged care assessor [4]. Most developed countries including England, Scotland, United States, Canada, Singapore, Japan, Sweden, Finland, France, Denmark, the Netherlands and Germany have a process which enables the assessment of an older person to determine their care needs and appropriate delivery of social and/or long-term care [5].

In Australia, care needs (including social, physical, medical, psychological and home and personal safety) are determined by the National Screening and Assessment Framework [6], conducted by ACATs, of which there are 80 nationally across all states and territories. The Australian government provides funding to state and territory governments to operationally administer ACATs [7], in which aged care assessors deliver assessment services on behalf of the Federal Minister for Health and Aged Care and the Australian Government Department of Health and Aged Care [8]. Operational responsibilities of states and territories include managing the timely delivery of assessments under the *Aged Care Act 1997*, and the management, training and performance of individual aged care assessors [7]. In essence, the pathway to government-funded aged care services in Australia begins with an older person applying for care, followed by a needs-based assessment conducted by ACATs to determine their eligibility for services. Once eligibility has been determined and access granted, government-funded aged care services are delivered by approved aged care service providers under the *Aged Care Act 1997*. The functions of ACATs and approved aged care service providers are separate, such that, ACATs determine eligibility for, and access to services, while approved aged care service providers deliver care and support services.

While the operation of ACATs is managed by states and territories, the Commonwealth Department of Health and Aged Care has oversight and responsibility for the Aged Care Assessment Program, within which ACATs sit, including monitoring and reporting of assessment service performance against key performance indicators [9]. The quality of assessment services is governed by the Department of Health and Aged Care according to the Aged Care Assessment Quality Framework [10], using a three-tiered approach (Table 1).

**Table 1:** Aged care assessment quality framework [9], three-tiered approach

Tier level	What does is measure?	How is it measured?
One	Quality and accuracy of data (information obtained at the time of the assessment)	Quality checks by the assessment against the National Screening and Assessment Form [6] and best practice principles
Two	After-the-fact quality audits, including client satisfaction with the assessment service received	10-question satisfaction survey with performance targets ranging between 85%-100% [10]
Three	Third-party audits (including sample checks by external auditors)	Review of performance reports against performance targets

The quality of residential aged care facilities (RACF) is also governed by the Department of Health and Aged Care, using the National Aged Care Quality Indicator Program. This program is mandatory and requires RACFs to collect and report on quality indicators (QIs) across the areas of pressure injuries, physical restraint, unplanned weight loss, falls and major injury, medication management, activities of daily living, incontinence care, hospitalisation, workforce, consumer experience and quality of life [11]. QIs for home care services were recommended by the Royal Commission into Aged Care Quality and Safety [12], and while this recommendation is viewed as a positive step toward measuring system performance, it fails to recognise the essential functions of ACATs which initially assess an older person’s eligibility to access government-funded aged care services. An older person’s journey through the Australian aged care system is not always linear. They may move between aged care types, and subsequently may need to undergo more than one assessment to determine their eligibility (for example when transitioning from hospital to home and requiring short term rehabilitation care or transitioning from the community to a residential aged care facility). In essence, to access any aged care type an assessment to determine eligibility is required in the first instance.

QIs can be used in various ways including documenting quality of care; making comparisons across time; setting priorities; supporting accountability; regulation and accreditation; and supporting quality improvement and a person’s choice of providers [13]. QIs can relate to the structure, process or outcomes of health care. Structure refers to attributes of the setting in which the care occurs, process refers to what is being done in the giving and receiving of care and outcomes describe the effects of care on the recipient’s health status [14]. PC-QIs have been defined as “the unit of measurement of the healthcare system, organisational, or individual performance, that quantifies patients’ and families’ experiences with the care received and the experience of an individual who needs to contact healthcare services”, a definition the Agency for Healthcare Research and Quality adapted to reflect the focus on the person and family [[15] (p.2)]. Whilst PC-QIs can be in any of these three formats, they are most commonly either process or outcome QIs.

The Australian government has committed to the provision of high-quality care for older Australians and as part of reforming the aged care system, it is developing an end-to-end system where the person drawing on the service drives quality [10]. For this to happen, it seems fitting that PC-QIs are used to measure system performance.

Twenty-four process PC-QIs measuring the quality of the aged care assessment service were developed in a previous study using a modified Delphi method [16]. This work was undertaken in collaboration with the evaluating Quality of Care (eQC) Patient and Carer Advisory Board, who were instrumental in assessing the person-centredness of the PC-QIs. The Board is a collaboration of patients and carers who support the embedding of partnerships between lived experience experts and researchers undertaking quality-of-care research. Members of the eQC Board were engaged in this study to provide their reflections on the research findings regarding the importance of PC-QIs for aged care assessment services, for current and future service users (Table 2).

**Table 2:** Members of the eQC Patient and Carer Advisory Board reflections on the importance of PC-QIs

Members of the eQC Patient and Carer Advisory Board agreed that a person-centred assessment process is essential to the delivery of an efficient and user-friendly service. From a person-centred viewpoint, members raise some important concerns for aged care assessment services to consider when conducting assessments. Whilst these concerns were raised by members of the Board only, they may also be shared by many older people and include: ‘Will the aged care assessment reflect my needs?’ and “Will the assessment consider my personal preferences about how to meet my needs?’ Understanding how these concerns influence the delivery of the aged care assessment service, go beyond the quality measures that currently exist for these services	
Including the person and their carer network, who are central to the aged care assessment process, in all discussions about the person’s care needs is essential. Not only does this provide the person an opportunity to voice their care needs but provides a platform for assessors to begin to understand the persons views and preferences about how to best meet their care needs. In addition, it provides the assessor with a foundational understanding of the person’s carer network, and how to support these networks to enable their sustainability in the long-term. This, however, can only be achieved if the person and their carer network, are provided information that is easy to understand, and which does not contain jargonistic language. On many occasions an abundance of information is provided during an assessment. This contributes to the already overwhelming assessment process and makes it more difficult for a person, and their carer network, to make informed decisions about how to best meet their needs. Furthermore, assessments are often ‘time limited’ which does not always support a person to clarify any questions they may have about their care needs during their assessment. This limited time, coupled with an abundance of information that is often jargonistic and difficult to understand, does not promote a person-centred approach to the assessment process. Rather, it places the person and their carer network at the periphery of the assessment process	

This paper presents the theoretical assessment confirming the consensus of PC-QIs for aged care assessment services. The objective of this study was to investigate the degree to which the PC-QIs reflected current and future service users’ perspectives of important quality measures using the RAND-UCLA Appropriateness Method to reach consensus, to reflect a person-centred approach. The Guidance for Reporting Involvement of Patients and the Public (revised) [17] (GRIPP2) short form (Appendix 1) outlines our reporting of public involvement in this study.

## Methods

This was a mixed methods study of 25 individual voting sessions conducted face-to-face or via telephone, depending on the participants preference. During the session, participants voted on the importance of each PC-QI using a five-point rating scale ranging [18] from 1 ‘extremely unimportant’ to 5 ‘extremely important.’ At the commencement of the session participants were advised they could provide verbal comments throughout. Sessions were audio recorded. Quantitative data from participant votes for each PC-QI were calculated and statistically described by median, interquartile range, consensus met, percentile, percentile rank, rank order, median and standard deviation. Quantitative data for each PC-QI were then assessed against the consensus statement for elimination and rank ordered according to importance to participants. Content analysis of qualitative data from audio transcriptions was conducted to determine the presence of certain words supporting participant votes for each PC-QI.

### Setting

This study was conducted in the homes of community-dwelling older people residing in the greater Brisbane area, Australia. Ranked one of the third largest metropolitan areas in Australia, the estimated resident population of Brisbane was 2.5 million in June 2022 and one of the fastest growing metropolitan areas in Australia [19].

### Ethical approval

This study was approved by the Royal Brisbane & Women’s Hospital Human Research and Ethics Committee (HREC/2022/QRBW/82417) and ratified by The University of Queensland research ethics and integrity unit (2022/HE001183). Governance approval was granted from Metro North Hospital and Health Service Community & Oral Health Directorate, Queensland.

### Recruitment of participants

A suggested representative sample of older people was determined using data from the 2020–2021 report on the Aged Care Act 1997 [2] and the Australian Institute of Health and Welfare Dementia in Australia 2021 report [20]. Participants were recruited by advertising the study using several strategies including word-of-mouth, information flyers, social media, public speaking engagements and carer support networks. Information flyers were included in appointment paperwork mailed out to prospective clients of the largest aged care assessment service in Queensland, and a community-based approach was adopted to facilitate greater participation in, and acceptance of the study by engaging with community groups and leaders of current and future aged care assessment service users [21]. Meetings were held with presidents and newsletter editors of six older peoples’ community groups, to provide an overview of the research study, enable opportunities to ask questions, and identify a suitable approach for advertising the study more broadly to members of each group. A presentation was delivered to members of Shed West Community Men’s Shed at their monthly meeting, where information about the research study was provided and an open forum was held to discuss pathways to access aged care services in Australia, including the role of aged care assessment services.

Information about the study was advertised in newsletters of relevant older people’s groups (e.g., Probus Club, Care of the Older Australian Queensland Branch, National Seniors Kenmore, and Shed West).

Facebook was the primary social media platform used to advertise the study. Twenty-eight people contacted the lead investigator, and 25 confirmed their interest to participate. A participant information sheet and consent form, which comprised of an accompanying easy read version (Appendix 2), an outline of the service elements of an aged care assessment (Appendix 3) and information on how an aged care assessment can help (Appendix 4), were mailed to interested participants.

### Participant voting sessions

Participant sessions were undertaken from February to July 2023. Voting sessions ranged from 60 to 90 min in duration. The capacity of each participant to consent to take part in the research was confirmed at the time of the session using the Evaluation to Sign Consent Measure. This tool assesses a person’s cognitive capacity to understand and consent to participate in research and has been validated for use with people living in residential aged care [22]. The tool can be tailored to a research protocol and asks participants to respond to five questions [23]. Questions asked of participants and the corresponding acceptable responses are detailed in Table 3.

**Table 3:** Evaluation to Sign Consent Measure questions and corresponding acceptable responses

Question	Acceptable responses
Can you name at least two potential risks about participating in this study?	I may become tired, exhausted, fatigued, distressed, upset
Can you tell me what is expected from you during this study?	Participate in one or two sessions, tell you how important I think a statement is to me, my age, my ethnicity
Can you tell me what you need to do if you don’t want to continue in this study?	Contact you (phone or email)
Can you tell me what you need to do if you experience any discomfort during the session today?	Tell you, ask for a break
Can you tell me how it was decided who can participate in this study?	My age, where I live

Participants were advised they could withdraw from the study at any time. Demographic data were collected from participants before the session commenced. A paper-based recording sheet outlining the 24 PC-QIs was provided to participants (Appendix 5), who were then asked to record their vote for each PC-QI on the sheet provided. Before voting commenced, participants were provided background information about the study and an opportunity to review the two information sheets that were previously sent in the mail (Appendices 3 & 4). Consent to record the session was provided verbally by participants. Participant voting sheets were collated after the session and the audio recordings of the sessions were subsequently transcribed verbatim.

### Data collection

Participants completed a paper-based data sheet with demographic data including their gender, age, ethnicity, previous involvement with an aged care assessment service, and primary medical diagnosis, including diagnosis of dementia and/or difficulties with memory. The quantitative data from participants’ voting sheets and the qualitative data from the audiotaped recordings of the sessions comprised the preliminary data for analysis. Following the session, participants were asked whether they would like to check their responses by subsequently reviewing their transcript (when available) and voting record sheet via email, hard copy in the mail or over the telephone.

### Data analysis

#### Quantitative data

Participant votes on each PC-QI were calculated (median; interquartile range; percentile; percentile rank; mean and standard deviation). This data was used to determine whether consensus was met, variation in the data and the rank order. The rank order was determined by prioritising the voting outcomes using the median as the comparator score to determine the percentile for each PC-QI, then calculating the percentile rank by applying the following formula (percentile rank = percentile ÷ 100 × [n ∔ 1]). Variation was determined by assessing the means and standard deviations.

The research team agreed on a consensus standard prior to voting sessions taking place. PC-QIs that did not meet the consensus standard would be eliminated. The criteria for the *a priori* consensus standard were: median of the PC-QI must be ≥ 3 on the 5-point scale (1 = extremely unimportant, 5 = extremely important); and interquartile range of the PC-QI must be ≤ 2.

#### Qualitative data

Content analysis of qualitative data from the transcriptions was conducted by the lead investigator [24]. The intent was not to derive themes from the qualitative data, but to add further insights to participants’ rating of each PC-QI. The transcripts were reviewed to identify if there was text which explained or clarified the voting choice of participants. Keywords were not searched for in the text, rather the priority was the concept of clarifying the vote choice. Relevant texts were highlighted, extracted, and categorised by PC-QI and participant identification code; then reviewed in batches according to each PC-QI. The qualitative data was used to understand whether the strength of the discussion supporting specific PC-QIs was similar to that of the ranking of the PC-QIs in the quantitative outcomes. The data was not quantified for analysis.

## Results

### Participants

Twenty-five people participated (face-to-face *n* = 15; telephone *n* = 10). Fifteen were recruited through information flyers mailed out by one aged care assessment service, five in response to newsletter advertisements, and five through advertisements at community-based carer support networks. There were 18 female participants (72%), and 24 were Caucasian (96%). All were aged between 66 to 90 years. Participants’ demographic characteristics are displayed in Table 4.

**Table 4:** Demographic data of participants

Participant	Age	Gender	Ethnicity	Last experience with an aged care assessment team	Primary medical diagnosis	Diagnosis of dementia	Difficulties with memory
CID-1	67	Female	Caucasian	2 years	Arthritis	No	No
CID-2	69	Male	Caucasian	Twice in last 14 months	Leukemia; peripheral neuropathy	No	No
CID-3	82	Female	Caucasian	14 years	Paraplegia	No	No
CID-4	84	Male	Caucasian	No experience	Orthopaedic fractures	No	No
CID-5	85	Female	Caucasian	10 months	Osteoporosis; multiple fractures	No	No
CID-6	74	Female	Caucasian	18 months	Arthritis; asthma	No	No
CID-7	75	Male	Caucasian	2 years	Cardiac disease; type II Diabetes Mellitus	No	No
CID-8	76	Female	Caucasian	No experience	Arthritis	No	No
CID-9	90	Female	Caucasian	1 year	Memory impairment; cardiac disease	No	Yes
CID-10	81	Female	Caucasian	6 years	Spinal stenosis	No	No
CID-11	78	Female	Caucasian	3 years	Kidney failure; heart failure; breast cancer; hip replacement; knee replacement	No	No
CID-12	81	Female	Caucasian	First experience scheduled for following day	Arthritis	No	No
CID-13	79	Female	Caucasian	6 years	Arthritis	No	No
CID-14	83	Female	Caucasian	1 week	Asbestosis; arthritis; bilateral peripheral neuropathy; bilateral carpel tunnel syndrome	No	Yes
CID-15	79	Female	Caucasian	8 months	Bronchiectasis; polio; left club foot	No	Yes
CID-16	69	Female	Caucasian	1 month	Schizophrenia; anxiety; bilateral peripheral neuropathy; bilateral carpel tunnel syndrome	No	Yes
CID-17	80	Male	Caucasian	No experience	Osteoarthritis	No	No
CID-18	88	Female	Caucasian	5 years	Osteoarthritis	No	No
CID-19	81	Female	Caucasian	No experience	Hypertension	No	No
CID-20	66	Female	Turkish	6 months	Osteoporosis; peripheral neuropathy, obesity; hypertension	No	No
CID-21	77	Female	Caucasian	4 years	Type II Diabetes Mellitus; scoliosis; spinal stenosis	No	No
CID-22	83	Male	Caucasian	2 weeks	Chronic heart failure	No	No
CID-23	87	Male	Caucasian	4 years	Chronic Obstructive Pulmonary Disease; prostate cancer; lymphoma	No	No
CID-24	69	Female	Caucasian	2 years	Osteoporosis; arthritis; multiple fractures	No	No
CID-25	79	Male	Caucasian	1 day	Parkinson’s disease; ankylosing spondylosis	No	No

*CID* Client Identifcation

### Capacity to consent

All participants displayed capacity to consent using the Evaluation to Sign Consent Measure [22]. Whilst two participants’ carers were present during the session, using this measure to establish if the participant could provide their own consent without the support of their carer, enabled their participation and inclusion of their opinions.

### Participants’ confirmation of results

All participants were asked to review their PC-QI record sheet and written transcript of the audiotaped session. Three out of 25 participants requested an amendment be made to their final vote (change of rating). In accordance with participants’ preferences, six participant responses were confirmed following telephone contact with the lead investigator, where results were presented and discussed, six were validated via hard copy sent in the mail, and 13 by email.

### Quantitative data

No PC-QIs were eliminated after the first round of participant votes (Table 5) in accordance with the consensus standard, however variation in the responses provided by participants was found and is displayed using the rank order of relative importance and standard deviation score for each PC-QI. Of the 24 PC-QIs, seven did not record any votes below four (1–5 scale). Furthermore, eight had a median of five (1–5 scale) indicating that overall, these PC-QIs were regarded as ‘extremely important’, and an interquartile range of zero, indicating there was no variability within the middle 50% of the data, with most participants voting similarly for these PC-QIs. Of the eight PC-QI’s, four represented the quality domain ‘respect for client’, three, ‘clear communication’ and one, ‘health care staff knowledge’.

**Table 5:** PC-QIs: Participant votes, median, interquartile range, consensus standard, percentile rank, rank order, median, standard deviation

Statement number	1		2	3	4	5	6	7	8	9	10	11	12	13	14	15	16	17	18	19	20	21	22	23	24
CID-1 vote	**5**		**5**	**5**	**5**	**5**	**5**	**5**	**5**	**5**	**5**	**5**	**5**	**5**	**5**	**5**	**5**	**5**	**5**	**5**	**5**	**5**	**5**	**4**	**5**
CID-2 vote	**5**		**4**	**1**	**5**	**4**	**5**	**5**	**1**	**5**	**4**	**4**	**4**	**4**	**4**	**4**	**4**	**4**	**5**	**1**	**5**	**5**	**5**	**3**	**4**
CID-3 vote	**5**		**4**	**4**	**3**	**3**	**4**	**4**	**4**	**4**	**5**	**5**	**4**	**3**	**4**	**4**	**3**	**4**	**4**	**4**	**5**	**4**	**4**	**5**	**4**
CID-4 vote	**5**		**4**	**5**	**4**	**5**	**4**	**5**	**3**	**5**	**4**	**4**	**4**	**5**	**3**	**3**	**5**	**4**	**4**	**2**	**5**	**5**	**2**	**2**	**5**
CID-5 vote	**5**		**5**	**4**	**5**	**5**	**4**	**5**	**4**	**5**	**4**	**5**	**5**	**4**	**4**	**4**	**5**	**5**	**5**	**3**	**5**	**5**	**5**	**5**	**5**
CID-6 vote	**5**		**5**	**5**	**4**	**5**	**5**	**5**	**5**	**5**	**5**	**5**	**5**	**5**	**5**	**5**	**5**	**5**	**5**	**5**	**5**	**5**	**5**	**5**	**5**
CID-7 vote	**5**		**5**	**5**	**5**	**5**	**5**	**5**	**5**	**5**	**5**	**5**	**5**	**4**	**5**	**5**	**4**	**5**	**5**	**5**	**5**	**5**	**5**	**5**	**5**
CID-8 vote	**5**		**5**	**5**	**5**	**5**	**5**	**5**	**5**	**5**	**5**	**5**	**5**	**5**	**5**	**5**	**5**	**5**	**5**	**5**	**5**	**5**	**5**	**5**	**5**
CID-9 vote	**5**		**5**	**5**	**5**	**5**	**5**	**5**	**5**	**5**	**5**	**5**	**5**	**5**	**5**	**5**	**5**	**5**	**5**	**5**	**5**	**5**	**5**	**5**	**5**
CID-10 vote	**5**		**5**	**4**	**5**	**4**	**5**	**5**	**5**	**5**	**5**	**5**	**5**	**5**	**5**	**5**	**5**	**5**	**5**	**5**	**5**	**5**	**5**	**5**	**5**
CID-11 vote	**5**		**5**	**3**	**5**	**3**	**5**	**5**	**5**	**5**	**5**	**5**	**5**	**5**	**5**	**5**	**5**	**5**	**5**	**4**	**5**	**5**	**5**	**5**	**5**
CID-12 vote	**5**		**5**	**4**	**5**	**4**	**5**	**5**	**4**	**5**	**5**	**5**	**5**	**5**	**5**	**5**	**5**	**5**	**4**	**5**	**5**	**5**	**5**	**5**	**5**
CID-13 vote	**5**		**4**	**4**	**4**	**4**	**5**	**5**	**5**	**4**	**4**	**5**	**5**	**5**	**4**	**5**	**5**	**5**	**5**	**4**	**5**	**5**	**5**	**5**	**5**
CID-14 vote	**5**		**5**	**5**	**5**	**4**	**5**	**5**	**5**	**5**	**5**	**5**	**5**	**5**	**5**	**5**	**5**	**5**	**5**	**5**	**5**	**5**	**5**	**5**	**5**
CID-15 vote	**5**		**5**	**5**	**5**	**5**	**5**	**5**	**5**	**5**	**5**	**5**	**5**	**5**	**5**	**5**	**5**	**5**	**5**	**5**	**5**	**5**	**5**	**5**	**5**
CID-16 vote	**5**		**4**	**5**	**3**	**5**	**3**	**3**	**5**	**5**	**2**	**5**	**5**	**2**	**4**	**5**	**3**	**2**	**2**	**5**	**4**	**5**	**5**	**5**	**3**
CID-17 vote	**4**		**4**	**3**	**4**	**3**	**5**	**5**	**3**	**4**	**4**	**4**	**5**	**5**	**5**	**5**	**5**	**5**	**4**	**3**	**5**	**5**	**5**	**5**	**5**
CID-18 vote	**5**		**5**	**5**	**5**	**5**	**5**	**5**	**5**	**5**	**5**	**5**	**5**	**5**	**5**	**5**	**5**	**5**	**5**	**5**	**5**	**5**	**5**	**5**	**5**
CID-19 vote	**5**		**5**	**5**	**5**	**5**	**5**	**5**	**5**	**5**	**5**	**5**	**5**	**5**	**5**	**5**	**5**	**5**	**5**	**5**	**5**	**5**	**5**	**5**	**5**
CID-20 vote	**5**		**5**	**5**	**5**	**5**	**5**	**5**	**5**	**5**	**5**	**5**	**5**	**5**	**5**	**5**	**5**	**5**	**5**	**5**	**5**	**5**	**5**	**5**	**5**
CID-21 vote	**5**		**5**	**5**	**5**	**5**	**5**	**5**	**5**	**5**	**5**	**5**	**5**	**5**	**5**	**5**	**5**	**5**	**5**	**5**	**5**	**5**	**5**	**5**	**5**
CID-22 vote	**5**		**5**	**4**	**4**	**5**	**4**	**5**	**4**	**5**	**5**	**4**	**5**	**5**	**5**	**5**	**5**	**5**	**4**	**4**	**5**	**5**	**5**	**5**	**5**
CID-23 vote	**5**		**5**	**5**	**5**	**5**	**5**	**5**	**5**	**5**	**5**	**5**	**5**	**5**	**5**	**5**	**5**	**5**	**5**	**5**	**5**	**5**	**5**	**5**	**5**
CID-24 vote	**5**		**5**	**5**	**5**	**5**	**5**	**5**	**5**	**5**	**5**	**5**	**5**	**5**	**5**	**5**	**5**	**5**	**5**	**5**	**5**	**5**	**4**	**5**	**5**
CID-25 vote	**4**		**5**	**3**	**5**	**4**	**5**	**5**	**3**	**4**	**5**	**5**	**5**	**5**	**5**	**4**	**5**	**5**	**4**	**3**	**5**	**5**	**5**	**4**	**5**
Median	5		5	5	5	5	5	5	5	5	5	5	5	5	5	5	5	5	5	5	5	5	5	5	5
CONSENSUS (Med) [GE 3]	Y		Y	Y	Y	Y	Y	Y	Y	Y	Y	Y	Y	Y	Y	Y	Y	Y	Y	Y	Y	Y	Y	Y	Y
1st Quartile	5		4.75	4	4.75	4	4.75	5	4	5	4.75	5	4.75	4	4	4	4.75	4.75	4.75	3.75	5	5	5	4.75	5
3rd Quartile	5		5	5	5	5	5	5	5	5	5	5	5	5	5	5	5	5	5	5	5	5	5	5	5
Interquartile range (IQR)	0		0.25	1	0.25	1	0.25	0	1	0	0.25	0	0.25	1	1	1	0.25	0.25	0.25	1.25	0	0	0	0.25	0
CONSENSUS (IQR) [LE 2]	Y		Y	Y	Y	Y	Y	Y	Y	Y	Y	Y	Y	Y	Y	Y	Y	Y	Y	Y	Y	Y	Y	Y	Y
# Scores < 5	2		6	10	7	9	5	2	8	4	6	4	3	5	7	5	4	4	7	9	1	1	3	4	3
Percentile	8%		24%	40%	28%	36%	20%	8%	32%	16%	24%	16%	12%	20%	28%	20%	16%	16%	28%	36%	4%	4%	12%	16%	12%
Percentile Rank	0.003		0.009	0.015	0.011	0.014	0.008	0.003	0.012	0.006	0.009	0.006	0.005	0.008	0.011	0.008	0.006	0.006	0.011	0.014	0.002	0.002	0.005	0.006	0.005
Rank order (1 = highest, 10 = lowest)	2		6	10	7	9	5	2	8	4	6	4	3	5	7	5	4	4	7	9	1	1	3	4	3
Mean	4.92		4.76	4.36	4.64	4.52	4.76	4.88	4.44	4.84	4.68	4.84	4.88	4.68	4.72	4.76	4.76	4.64	4.64	4.64	4.96	4.96	4.8	4.72	4.84
Standard deviation	0.28		0.44	1.01	0.63	0.69	0.52	0.44	1.0	0.37	0.69	0.37	0.33	0.75	0.54	0.52	0.61	0.66	0.7	1.15	0.21	0.21	0.65	0.74	0.47
*CID* Client Identification																									
*Legend*																									
Statement number		PC-QI – Title and Descriptor																							
1		**Dignity during booking** At the time of booking my assessment, I was treated with dignity and respect																							
2		**Equitable booking process** At the time of booking my assessment, I was treated as an equal partner in the decision-making process																							
3		**Respect during booking** At the time of booking my assessment, my cultural and/or religious preferences were respected																							
4		**Appointment convenience** My assessment appointment was scheduled at a time that was convenient to me																							
5		**Informed about support person** I was advised I could have a support person attend my assessment appointment if I so desired																							
6		**Health Care Staff Knowledge** During my assessment interview, I received information about the aged care assessment process which gave me confidence in the Health Care Staff’s knowledge																							
7		**Health Care Staff clarity** During my assessment interview, I could understand what the assessor said to me																							
8		**Respect during assessment interview** During my assessment interview, my cultural and/or religious preferences were respected																							
9		**Dignity during assessment interview** During my assessment interview I was treated with dignity and respect																							
10		**Support during assessment interview** During my assessment interview, I was supported to raise any concerns about getting the help I need																							
11		**Equitable assessment interview** During my assessment interview, I was treated as an equal partner in the care planning process																							
12		**Involved in decisions at interview** During my assessment interview, I was provided with opportunities to make decisions about my care needs																							
13		**Adequate time for discussion** During my assessment interview, I had enough time to talk with the assessor																							
14		**Adequate time for decision-making** During my assessment interview, I had enough time to make decisions																							
15		**Responsibilities of Aged Care Assessment Team explained** During my assessment interview, the assessor explained the responsibilities of the Aged Care Assessment Team																							
16		**Clear explanations by assessor** After my assessment interview was completed, the assessor explained what the next steps were																							
17		**Knowledge of next steps** After my assessment interview was completed, the assessor explained what I was expected to do next																							
18		**Respectful written information** Written information I received explaining my care needs reflected dignity and respect																							
19		**Appropriate written information** Written information I received acknowledged the importance of cultural and spiritual preferences																							
20		**Accuracy of support plan** The support plan summary I received reflected my needs																							
21		**Contact information provided** If my care needs change after my assessment has been completed, I know who I can ask for assistance																							
22		**Eligibility of care explained** I know what care I am eligible to access																							
23		**Access to care explained** The assessor explained that an Aged Care Assessment delegate would decide what Aged Care Assessment type I would be approved to access																							
24		**Easy to understand written information** Information I was given at my assessment interview was easy to understand																							

Standard deviation scores across the 24 PC-QIs demonstrated variation in voting (ranging from 0.21 to 1.15). The six PC-QIs that showed the greatest variation, with a standard deviation score > 0.7 were: respecting a client’s cultural and/or religious preferences when booking an assessment; respecting a clients cultural and/or religious preferences during the assessment; providing the client adequate time to speak with the assessor; ensuring written information provided to a client about their care needs reflected dignity and respect; providing a client with written information that acknowledged spiritual and cultural preferences; and, explaining to the client an aged care delegate would decide what care type they were approved to access. Out of these six PC-QIs, three had a standard deviation of ≥ 1.0 which included: respecting a client’s cultural and/or religious preferences when booking an assessment; respecting a client’s cultural and/or religious preferences during an assessment and providing a client with written information that acknowledged spiritual and cultural preferences. Six PC-QIs had a standard deviation of ≤ 0.40 demonstrating the least variation and included: treating the client with dignity and respect at the time of booking the assessment; treating the client with dignity and respect during the assessment; treating the client as an equal partner in the care planning process during the assessment; providing the client with opportunities to make decisions about their care needs during the assessment; providing the client with an accurate support plan that reflected their needs and; advising the client who they can ask for assistance if their care needs change after the assessment has been completed.

### Qualitative data

After all the transcripts were reviewed, statements that aligned with each PC-QI were identified. Table 6 details these results and includes the five quality domains, PC-QIs that sit within each quality domain and the corresponding rank order determined by quantitative analysis. The qualitative findings confirmed the quantitative ranking order results.

**Table 6:** Qualitative data from participant interviews supporting rank order (1 = highest, 10 = lowest) of PC-QIs

Quality Domain	Number, title and descriptor	Rank order (1 – 10)	Qualitative data
**Health Care Staff Knowledge**	**[6] Health Care Staff Knowledge** During my assessment interview, I received information about the aged care assessment process which gave me confidence in the Health Care Staff’s knowledge	5	**CID-1** ‘*Being given accurate knowledge of which services are available is what would give me confidence. I think you need time to reflect on what has happened at the time of the assessment. You can’t answer this straight away. If you’re stressed it’s very important not to feel like there is a power imbalance between you and the assessor. You should be given all information and not just sufficient information which suits the system that It’s serving.’*
	**[7] Health Care Staff clarity** During my assessment interview, I could understand what the assessor said to me	2	**CID-15** ‘*They (assessors) just presume you have the same knowledge as them. They use all these different words and acronyms that I wouldn’t even know what they are talking about half the time. It is really like they are speaking a different language. They have to remember that we don’t have the knowledge they have, and that is the whole purpose of them coming to help us.’*
**Respect for Client**	**[3] Respect during booking** At the time of booking my assessment, my cultural and/or religious preferences were respected	10	**CID-2** ‘*I have a different culture to the majority, and I know I really don’t have any religious preferences, so this is pretty low for me.’* **CID-13** ‘*I don’t have religious preferences, but if I was Muslim and I didn’t want someone coming to my home on a Friday, that should be respected.’*
	**[8] Respect during assessment interview** During my assessment interview, my cultural and/or religious preferences were respected	8	**CID-6** *‘Well, I don’t think that assessors understand what spiritual preferences are. They talk about this as if it is like people going to the football. They don’t understand it is essential to one’s being. We are living in a secular society; your spiritual life is seen as being peripheral to their concerns.’*
	**[1] Dignity during booking** At the time of booking my assessment, I was treated with dignity and respect	2	**CID-13** ‘*It is very important to me because otherwise I wouldn’t even bother doing it. If people were blank, sadistic or sharp, or anything like that, I wouldn’t even go through with it.’*
	**[9] Dignity during assessment interview** During my assessment interview I was treated with dignity and respect	4	**CID-15** ‘*Doesn’t everybody deserve to be treated with dignity and respect? I would have thought that was required by a health professional.’*
	**[10] Support during assessment interview** During my assessment interview, I was supported to raise any concerns about getting the help I need	6	**CID-11** *“Absolutely. I am not just a number. I am a person. And not only a person, but an individual person.’* **CID-18** *“You certainly don’t want a power imbalance. That would definitely not encourage or support me to raise my concerns. I would probably do the opposite. You can’t feel like your concerns are being squashed, not recognised and not understood.’*
	**[2] Equitable booking process** At the time of booking my assessment, I was treated as an equal partner in the decision-making process	6	**CID-10** ‘*You want to be treated like a person and not a number.’*
	**[11] Equitable assessment interview** During my assessment interview, I was treated as an equal partner in the care planning process	4	**CID-4** ‘*The system directs the direction of what the assessor asks. Not the person. There is really no input from the person about what direction they think they should be taking, or how they look at their personal needs. The process is more predetermined by a set of needs that other people have experienced and not you as in individual.’* **CID-18** *‘There needs to be an equal partnership in decision making. If you don’t get the choice or option to make decisions, then there is no equal partnership.’*
	**[18] Respectful written information** Written information I received explaining my care needs reflected dignity and respect	7	**CID-23** ‘*Of course. I would have thought that it should never be an option, just a given.’*
	**[20] Accuracy of support plan** The support plan summary I received reflected my needs	1	**CID-16** ‘*If it doesn’t make sense and is not true, then why do they (assessor) bother even writing one.’*
	**[19] Appropriate written information** Written information I received acknowledged the importance of cultural and spiritual preferences	9	**CID-22** ‘*It is probably not critically important to me, but I don’t have any cultural or spiritual preferences. I can see that this would be more important to someone who had stronger preferences than me.’*
**Person centred approach**	**[4] Appointment convenience** My assessment appointment was scheduled at a time that was convenient to me	7	**CID-9** *‘I am hopeless in the morning because I have to get up and down at night. I don’t get around like I used to, and I need help in the shower now, so I couldn’t manage an appointment in the morning.’* **CID-14** *“Convenient is important. But you can’t expect to get the exact time, you have to be a bit flexible. But if you could work out a time between the two of you that would be best.’*
	**[5] Informed about support person** I was advised I could have a support person attend my assessment appointment if I so desired	9	**CID-7** ‘*That is definitely important. I would need my daughter to attend my assessment to help me understand what they are doing.’*
**Collaborative partnership with client**	**[12] Involved in decisions at interview** During my assessment interview, I was provided with opportunities to make decisions about my care needs	3	**CID-6** ‘*We need to know what is possible, not just what is available. We need to know and understand what possibilities are first. This needs to be explored in the first instance before you can make an informed decision.’* **CID-10** *‘As far as I am concerned, I’m the one that should be making the decisions.’*
	**[13] Adequate time for discussion** During my assessment interview, I had enough time to talk with the assessor	5	**CID-14** ‘*Sometimes you just need time to think about things, you don’t want to feel rushed. These are important decisions.’*
	**[14] Adequate time for decision-making** During my assessment interview, I had enough time to make decisions	7	**CID-1** ‘*It was only in the next maybe 10 days or so that I realised that I needed the processing time to be able to answer any questions and make decisions. You’re not in a position of power (as the client). You’re going to get something, and because of the way you are approached during the interview, that is the way it ends up appearing on paper. You might feel like what you really want is not legitimate, it’s not validated. It is like you are a little fish in a big pond.’*
**Clear communication**	**[15] Responsibilities of Aged Care Assessment Team explained** During my assessment interview, the assessor explained the responsibilities of the Aged Care Assessment Team	5	**CID-15** ‘*You have so many people coming into your house …you have to know who they are and what they do.’*
	**[16] Clear explanations by assessor** After my assessment interview was completed, the assessor explained what the next steps were	4	**CID-13** ‘*That is really important. If they never tell you that, you end up just sitting and waiting…..not knowing.’*
	**[17] Knowledge of next steps** After my assessment interview was completed, the assessor explained what I was expected to do next	4	**CID-10** ‘*It would be much better if I had some explanation about what was expected of me.’* **CID-13** *‘If I have to do anything then I need to know. I wouldn’t have a clue otherwise, and that is not much help. They (assessors) are meant to be assisting us, not hindering us.’*
	**[21] Contact information provided** If my care needs change after my assessment has been completed, I know who I can ask for assistance	1	**CID 7** ‘*it is about having control. If you don’t know which way to go, or what your can do, then you don’t have control and that is the problem. If I had a sore throat, I would know to call my doctor and they would tell me what to do*, *and if I didn’t get better I would go back. But you can’t do this with this process, because you don’t know who to go back to.’*
	**[22] Eligibility of care explained** I know what care I am eligible to access	3	**CID-5** ‘*Yes of course that is important. You need to know what care you are eligible for, but it’s very hard to find any information about that really.’*
	**[23] Access to care explained** The assessor explained that an Aged Care Assessment delegate would decide what Aged Care Assessment type I would be approved to access	4	**CID-13** ‘*you mean that my assessment goes somewhere, and then a different person makes a decision on my assessment? I should be told that….yes….that is really important.’*
	**[24] Easy to understand written information** Information I was given at my assessment interview was easy to understand	3	**CID-5** *‘I get so much information that I don’t understand that I just put in the bin. So it all just goes in the bin because it makes no sense to me. Unless someone is going to sit with me and explain it, then it doesn’t mean anything to me.’* **CID-15** *‘there is no point getting it otherwise.’*

*CID* Client Identification

## Discussion

To the best of our knowledge, this is the first study that defines a set of PC-QIs for Australian aged care assessment services using a consensus voting process that included current and future service users. The purpose of this was to ensure that the final set of PC-QIs better reflected what this group value as being of greatest importance when undergoing an assessment that determines their eligibility for, and access to, government-funded aged care services, confirming their person-centredness. Twenty-four evidence-based PC-QIs were quantitatively explored with participants, who voted on their perceived importance. All 24 PC-QIs met consensus on the first round of voting, indicating all were important to participants. While no PC-QIs were eliminated, variation within the ratings of some PC-QIs was observed. Qualitative data from audio recordings of participant voting sessions helps to explain the quantitative findings.

Involving people in research is becoming more important, with an increasing awareness of the value of involving them in the design, implementation and dissemination of health-related research [25, 26]. Furthermore, involving people at the centre of care is a pre-requisite for person-centred health care and has been shown to result in equitable healthcare solutions and improved health outcomes [26]. One of the major objectives of person-centred care is the establishment of a working relationship between the person, their family and service delivery [27]. The inclusion of people in research with diversity such as a wide age range, is emerging internationally as an ethical imperative [28]. Including older people and communities in research about person centred aged care services has been shown to improve physical and social well-being outcomes for older people, with co-design being the gold standard [29]. The objective of this study was to reach consensus on PC-QIs for aged care assessment services from the end user’s perspective. The PC-QIs presented were confirmed by placing those people who may need to access aged care assessment services at some point in their lifetime, at the centre of the voting process to establish a relationship between the person, their family and the aged care assessment process. In addition, members of the eQC Patient and Carer Advisory Board who were involved in the development of the preliminary PC-QIs to assess their person-centredness, were engaged to provide their reflections on the research findings on the importance of PC-QIs for aged care assessment services, for this user group (Table 2).

This study highlighted two PC-QIs voted by participants to be of highest priority for aged care assessment services. These were: the provision of a support plan summary that reflected their needs, and, providing them with information about who to contact if their care needs change. The second highest priorities for participants were treating them with dignity and respect when booking their assessment and being able to understand the assessor during their assessment interview.

While it was voted as important to participants, respecting cultural and/or religious preferences at the time of booking an assessment, undertaking an assessment, and acknowledging spiritual and cultural preferences when providing written information demonstrated the greatest variation across responses. This suggests that the opinions of some participants in this study about cultural and spiritual preferences differs.

Similar findings were reported in a study that developed PC-QIs through co-design for primary care services in Alberta, Canada. Participants in that study prioritised five PC-QIs including involving patients in decisions about their treatment and care; a trusting relationship with health care provider; health information to support person-centred care; co-designing care in partnership with communities and overall experience [30].

QIs have been shown to assist clinicians, organisations, and policy makers by providing a quantitative basis to monitor the care and processes by which care is delivered to people [31]. PC-QIs ensure a person’s perspective is reflected, and that what is measured reflects what is most important to the person when receiving the care and services they need [15]. Through the inclusion of the perspectives of current and future service users, regarding what they believe to be important when delivering aged care assessment services, the 24 PC-QIs presented in this study provide quantitative measures that Australian aged care assessment services can use to monitor the quality of the services they provide to ensure it is person-centred. PC-QIs have been shown to assist with the standardisation of collecting and reporting of data at a systems level enabling actionability to influence change, and as such, can be used to monitor system performance, and evaluate policy and practice in relation to person-centred care [32]. The quality of aged care assessment services is governed by the Australian Government Department of Health and Aged Care in accordance with the Aged Care Assessment Quality Framework [10], and as such, is monitored at a systems level, rather than at the provider level.

A Quality of Care Experience in Aged Care Instrument was developed to routinely measure the quality of care experienced by older people in home and residential aged care settings to support the routine measurement of the quality of care experiences [33, 34]. Patient experience measures are often used to measure performance at the provider level with little evidence of their use for system-level applications [30]. The 24 PC-QIs presented in this study support system-level applications and actionability to monitor aged care system performance related to eligibility and access to government-funded aged care services, while also including the service users’ voice.

The PC-QIs presented have applicability for aged care systems internationally. Many countries such as England, Scotland, United States, Canada, Singapore, Japan, Sweden, Finland, France, Denmark, the Netherlands and Germany, provide formal government funded-approved aged care for older people that is delivered by lead agencies in accordance with legislation that defines eligibility and access requirements and includes assessment of an older person’s needs [5]. Research findings presented in this study are relevant to understanding people’s views on what they perceive are quality assessment services that determine their care needs and eligibility to access appropriate aged care services, and bear relevance to those countries which undertake such assessments of older people.

Whilst a strength of this study is the involvement of older people who are current or future service users, one limitation of the study is that older people living in states and territories other than Queensland or in regional and remote areas of Queensland were not included. Another limitation is the low number of participants with a diagnosis of dementia and/or cognitive impairment, people from culturally and linguistically diverse backgrounds and those who identify as First Nations people. It is plausible that these groups may have different expectations regarding the quality of ACAT services and in recognition of this limitation, future work is required to explore the validity of these 24 PC-QIs in these population groups and for people living in other areas of Australia including regional and remote areas.

## Conclusion

The 24 evidence-based PC-QIs presented in this study addresses the gap in PC-QI development for the assessment component of the Australian aged care system. The evidence-based PC-QIs confirmed by consensus voting by older people form the theoretical assessment of content validity. There is an opportunity for the Australian government to pilot test the 24 evidence-based PC-QIs to enable assessment of construct validity to support their operationalisation. This will assist the Australian government in beginning to move towards the standardisation of data collection and reporting across the assessment component of the Australian aged care system that enables the evaluation of policy and practice in relation to person-centred care. Of the 24 PC-QIs, seven did not receive any votes below four (1–5 scale). Furthermore, eight PC-QIs had a median of five and interquartile range of zero. Out of the 24 PC-QIs, five did not receive any votes below four, had a median of five, and an interquartile range of zero. It is therefore recommended that any data set should include these five PC-QIs at a minimum. These five PC-QIs represented the quality domains respect for client (*n* = 4), and clear communication (*n* = 1). There is an opportunity for the Australian government to move toward a comprehensive program that measures the quality of the Australian aged care system from eligibility through to delivery of care, through the inclusion of these PC-QIs in the current suite of quality measures. Additionally, the 24 PC-QIs presented support the objectives of the Australian Government regarding the provision of aged care services which include, appropriately meeting the needs of older Australians including being that of being person-centred.

### Supplementary Information

Supplementary Material 1.

Supplementary Material 2.

Supplementary Material 3.

Supplementary Material 4.

Supplementary Material 5.

## Data Availability

Data are available upon reasonable request from the authors.

## Abbreviations

ACAT: Aged Care Assessment Team
eQC: evaluating Quality of Care
GRIPP2: Guidance for Reporting Involvement of Patients and the Public (revised)
PC-QIs: Person Centred Quality Indicators
QIs: Quality Indicators
RACF: Residential Aged Care Facility
