# Supplementary material for: Person-centred quality indicators for Australian aged care assessment services: a mixed methods study
Source: Res Involv Engagem. 2024 Aug 14;10:88. doi: 10.1186/s40900-024-00606-x (PMC11323374; doi:10.1186/s40900-024-00606-x)
Supplement: Supplementary file 1 — Supplementary Material 1. [file 40900_2024_606_MOESM1_ESM.docx]

**Appendix A: GRIPP2 Reporting Checklist – Short Form**

| **Section and topic** | **Item** | **Section where reported and details** |
| --- | --- | --- |
| 1: Aim | Report the aim of the PPI (patient and public involvement) in the study | Study aim is included in the background and in the methods section: *There are no measures that tell us if the aged care assessment service is of high quality from the perspective of the person being assessed. Quality measures have been developed and introduced in Australian residential aged care facilities. These however, have not considered the perspectives of those living in this setting. Quality measures for home care services have also been recommended.*  *This research aims to fill the gap in person-centred quality measures by asking current and future service users of aged care assessment services to vote on the importance of 24 person-centred quality indicators (PC-QIs), that were developed in a previous study using a modified Delphi method approach supported by engagement with a consumer led advisory board.* |
| 2: Methods | Provide a clear description of the methods used for the PPI in the study | The methods section outlines PPI in the development of the person-centred quality indicators. Participants recruited were current and/or previous service users of aged care assessment services. Recruitment approach included engagement with community groups through open forum invitations and newsletter advertisements. Public contributors are co-authors of this paper. The eQC Patient and Carer Adviory Board were involved in the development of the PC-QIs and were engaged in this study to provide their reflections on the voting preferences of older people regarding the importance of each PC-QI. |
| 3: Study Results | Outcomes – Report the results of the PPI in the study, including both positive and negative outcomes | *Table 4* details quantitative data and *Table 5* shows supporting qualitative data outlining results of consumer participation in the study. |
| 4: Discussion and conclusions | Outcomes – comment on the extent to which PPI influenced the study overall. Describe positive and negative effects | This is detailed throughout the discussion section: *this is* *the first study that defines a set of PC-QIs for Australian aged care assessment services using a consensus voting process that included current and potential future service users. The purpose of this was to ensure that the final set of PC-QIs better reflected what this group value as being of greatest importance when undergoing an assessment that determines their eligibility for, and access to, government-funded aged care services, confirming* *their person-centredness.* |
| 5: Reflections/critical perspective | Comment critically on the study, reflecting on the things that went well and those that did not, so others can learn. | Discussion section, Box 1, details PPI reflection on research results.  Strengths and limitations are included in the discussion section and discuss limitation of diversity of participants recruited including geographical location, cultural status, and medical diagnosis. |
