# Supplementary material for: Person-centred quality indicators for Australian aged care assessment services: a mixed methods study
Source: Res Involv Engagem. 2024 Aug 14;10:88. doi: 10.1186/s40900-024-00606-x (PMC11323374; doi:10.1186/s40900-024-00606-x)
Supplement: Supplementary file 2 — Supplementary Material 2. [file 40900_2024_606_MOESM2_ESM.docx]

**Easy read research information sheet**

This sheet is about the research you have agreed to participate in.


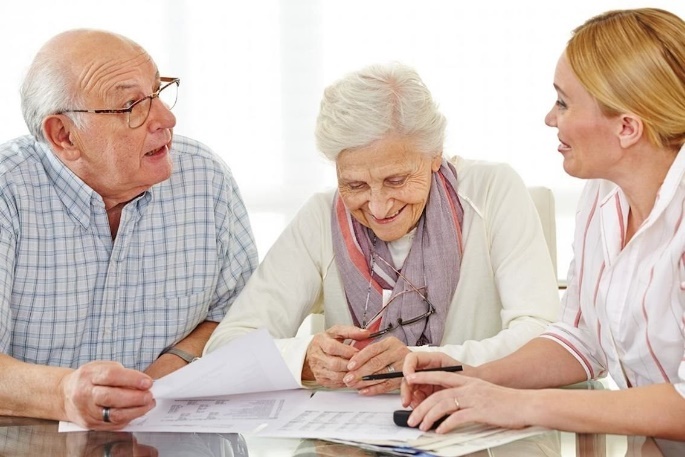


This research is about the aged care assessment process you may or may not have recently experienced. It is about what you think is important about that experience.


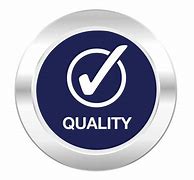


Quality is about receiving a good service.

It is important for the Aged Care Assessment Team to understand what you think a quality service is.


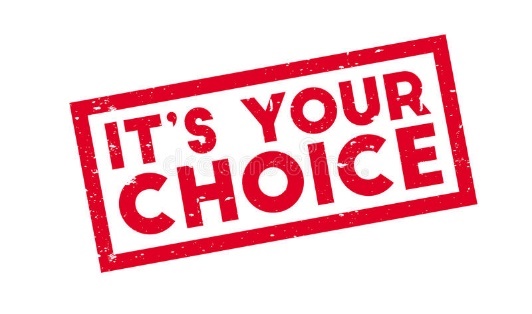


It is your choice if you would like to participate in this research.

*
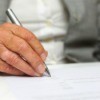
*

If you are happy to participate in this research, you will be asked to sign a form.


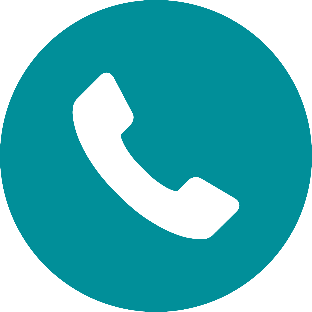


A researcher will call you to arrange a time to visit you at your house.


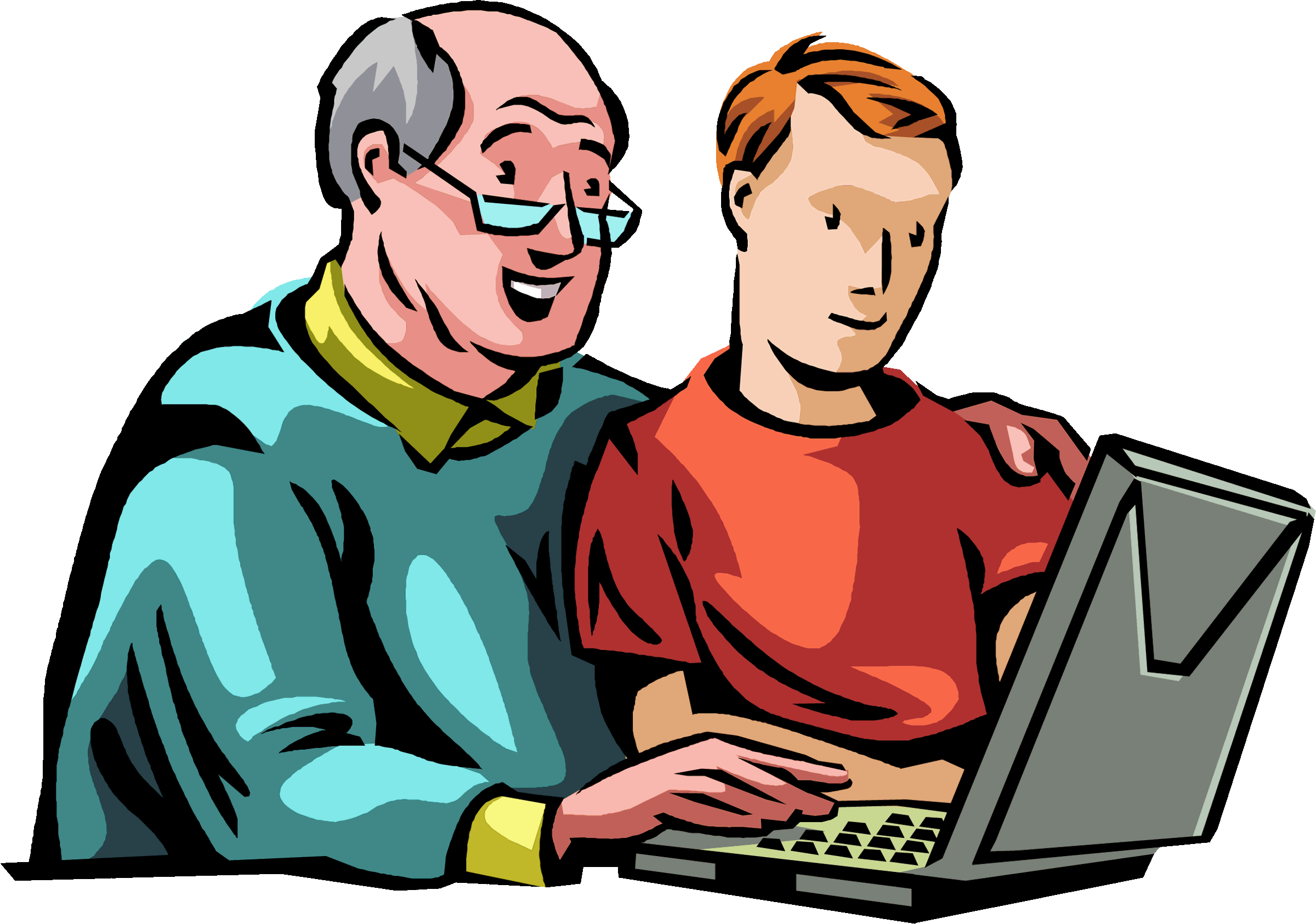


A member of your family, or somebody who supports you to make decisions can be with you to help you answer the research questions.


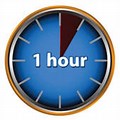
The visit at your house will take approximately one hour.

You can take as many breaks as you like if you get tired.


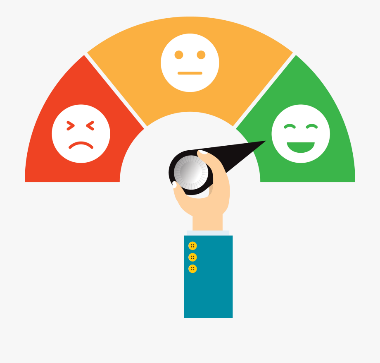


You will be asked some questions about what you think makes an aged care assessment a good experience for you.


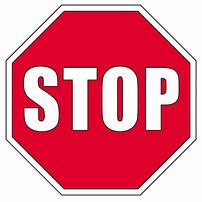
If you want to stop participating in the research at any time, you can.
