# Supplementary material for: Person-centred quality indicators for Australian aged care assessment services: a mixed methods study
Source: Res Involv Engagem. 2024 Aug 14;10:88. doi: 10.1186/s40900-024-00606-x (PMC11323374; doi:10.1186/s40900-024-00606-x)
Supplement: Supplementary file 3 — Supplementary Material 3. [file 40900_2024_606_MOESM3_ESM.docx]

**THE AGED CARE ASSESSMENT PROCESS**

1.
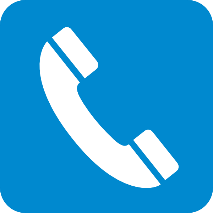
**REFERRAL REQUEST**

You, your family member, your doctor, a friend, or a health professional can request an aged care assessment for you.

**2.0** **ARRANGING YOUR ASSESSMENT TIME**


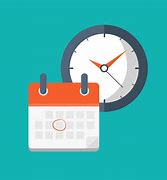
The administration staff from the Aged Care Assessment Team contact you, and/or your family member to arrange a day and time for the assessment to take place.

**3.0** **ON THE DAY OF THE** **AGED CARE ASSESSMENT**

An aged care assessor comes to your house and asks you some questions about what help you need. If restrictions are in place because of COVID 19, the assessor may call you instead. Your family member or a friend can be with you to help you answer the questions.
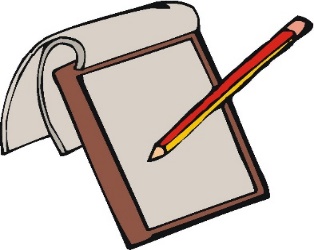


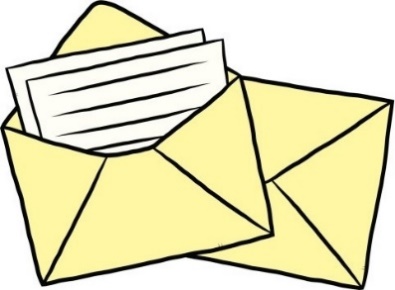


**4.0 RECEIVING THE SUPPORT PLAN SUMMARY**

Information is sent in the mail to you and/or a member of your family. This information tells you about what help you needed with your everyday activities.


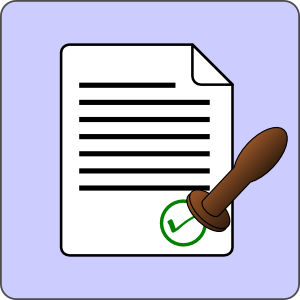
**5.0 APPROVAL LETTER**

A letter is sent in the mail to you and/or a member of your family. This letter gives you information on what government funded aged care services you were approved to access.
