# Supplementary material for: Person-centred quality indicators for Australian aged care assessment services: a mixed methods study
Source: Res Involv Engagem. 2024 Aug 14;10:88. doi: 10.1186/s40900-024-00606-x (PMC11323374; doi:10.1186/s40900-024-00606-x)
Supplement: Supplementary file 4 — Supplementary Material 4. [file 40900_2024_606_MOESM4_ESM.docx]

**How can the Aged Care Assessment Team help you?**

THE AGED CARE ASSESSMENT TEAM **GIVE YOU INFORMATION** ABOUT WHAT CARE IS AVAILABLE

The Assessment can take place:

1. **at your home,**
2. **in a hospital, OR**
3. **in a residential aged care facility**

**Access** to receive services under the Commonwealth Home Support Program

**Access** to receive the Short Term Restorative Care Program (time limited rehabilitation program provided in your home environment)

THE AGED CARE ASSESSMENT TEAM CAN **RE-ASSESS** YOUR CARE NEEDS IF YOUR CIRCUMSTANCES CHANGE.

THE AGED CARE ASSESSMENT TEAM **DETERMINE** IF YOU ARE **ELIGIBILE** TO **ACCESS AGED CARE**

**Access** to move into a Residential Aged Care Facility *permanently*

**Access** to move into a Residential Aged Care Facility *temporarily* (respite)

**Access** to receive a Home Care Package

THE AGE CARE ASSESSMENT TEAM **APPROVE** YOUR **ACCESS** TO AGED CARE
