# Supplementary material for: Person-centred quality indicators for Australian aged care assessment services: a mixed methods study
Source: Res Involv Engagem. 2024 Aug 14;10:88. doi: 10.1186/s40900-024-00606-x (PMC11323374; doi:10.1186/s40900-024-00606-x)
Supplement: Supplementary file 5 — Supplementary Material 5. [file 40900_2024_606_MOESM5_ESM.docx]

**RATING SCALE**

**On a scale of 1 to 5, where 1 is extremely unimportant and 5 is extremely important, how would you rate the importance of this statement?**

| **PUT A CIRCLE AROUND THE NUMBER THAT TELLS ME HOW IMPORTANT THIS STATEMENT IS TO YOU** |
| --- |
| At the time of booking my assessment, I was treated with dignity and respect  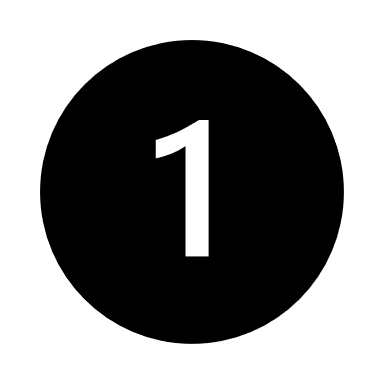  **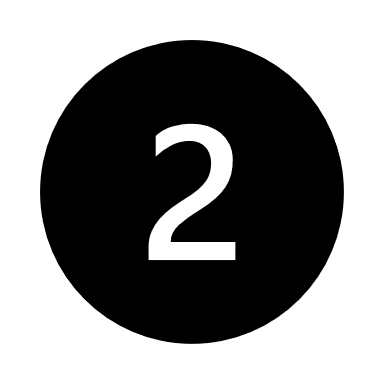** 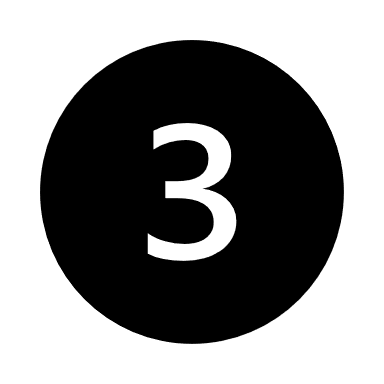 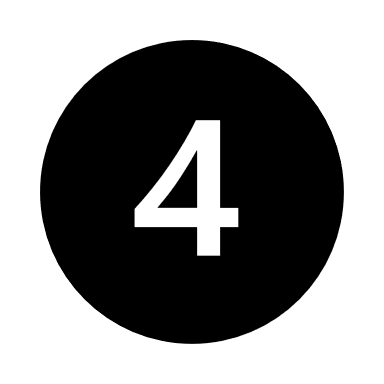 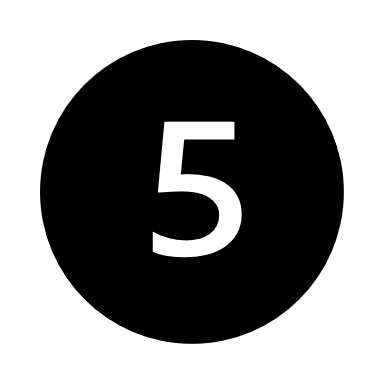  Extremely Extremely  Unimportant Important |
| At the time of booking my assessment, I was treated as an equal partner in the decision-making process  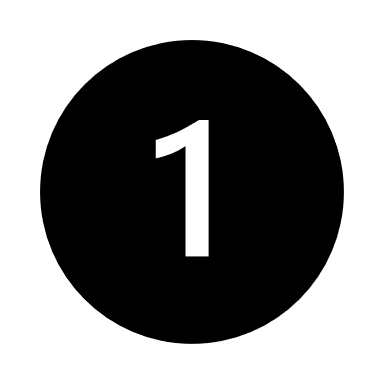  **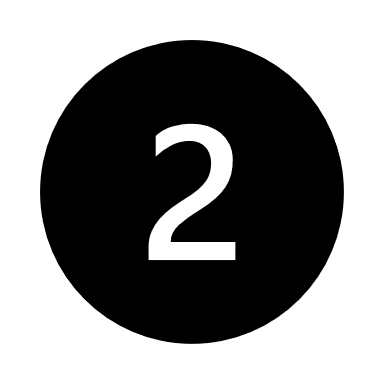** 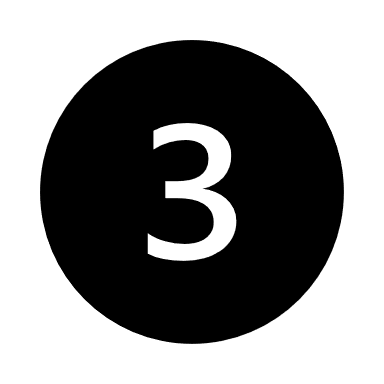 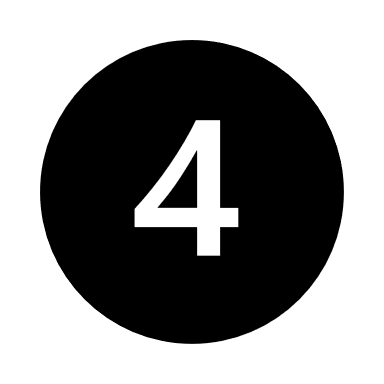 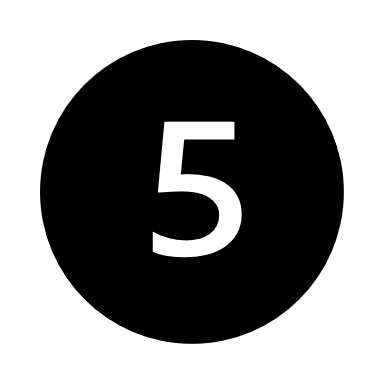  Extremely Extremely  Unimportant Important |
| At the time of booking my assessment, my cultural and/or religious preferences were respected  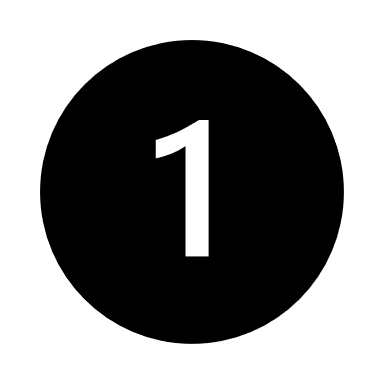  **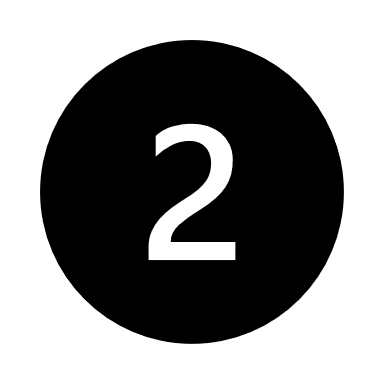** 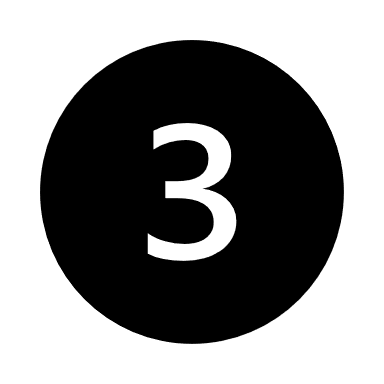 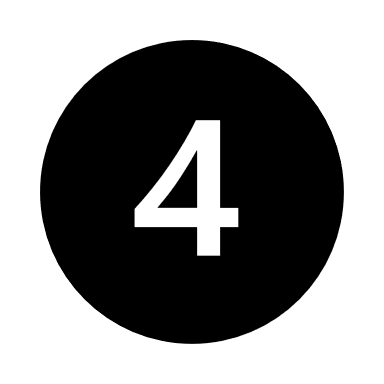 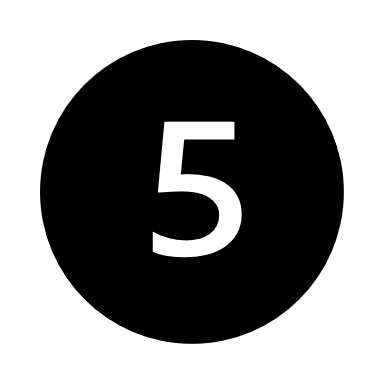  Extremely Extremely  Unimportant Important |
| My assessment appointment was scheduled at a time that was convenient to me  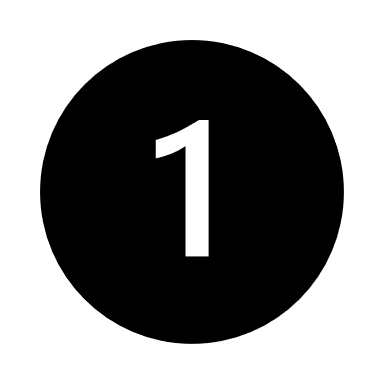  **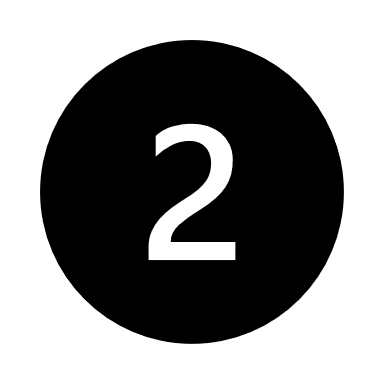** 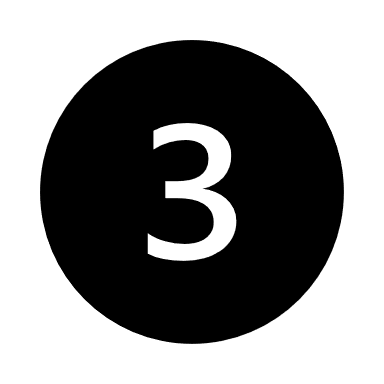 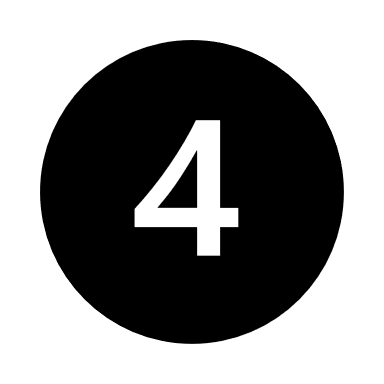 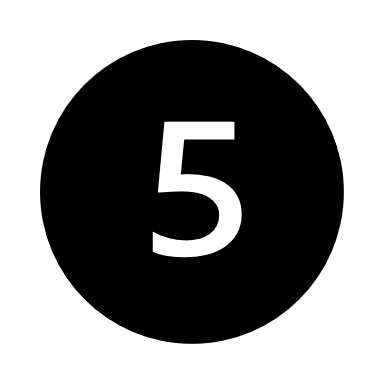  Extremely Extremely  Unimportant Important |
| I was advised I could have a support person attend my assessment appointment if I so desired  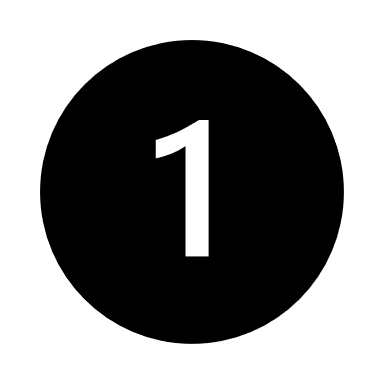  **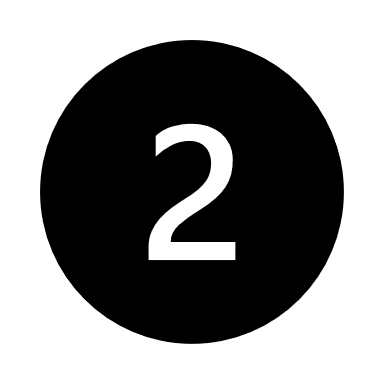** 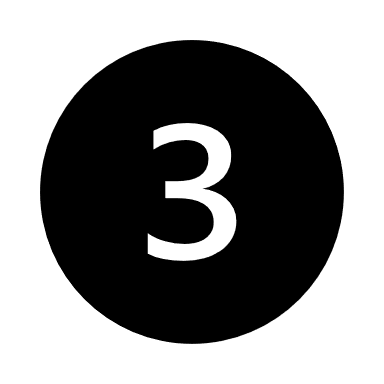 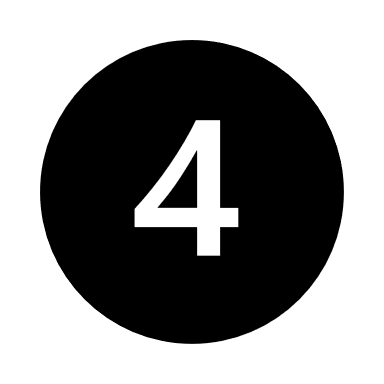 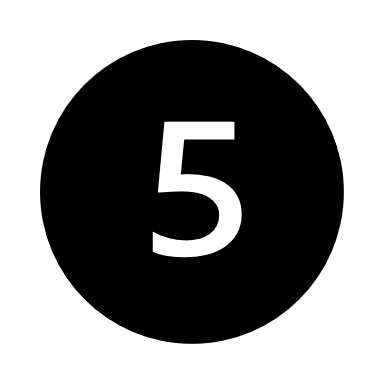  Extremely Extremely  Unimportant Important |
| During my assessment interview, I received information about the aged care assessment process which gave me confidence in the Health Care Staff’s knowledge  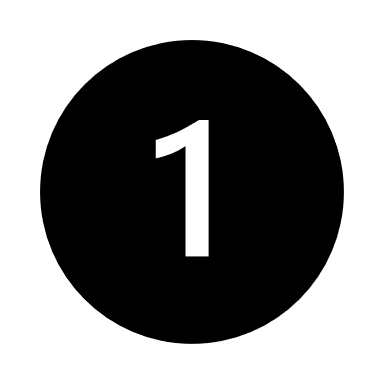  **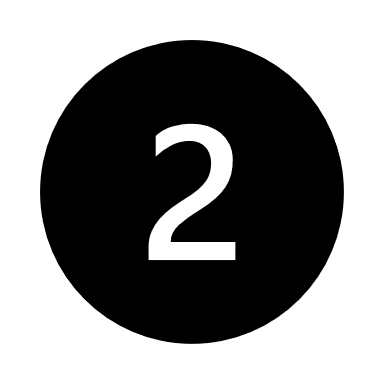** 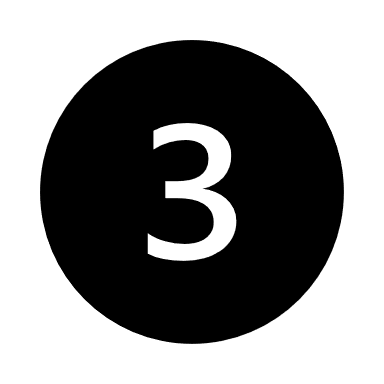 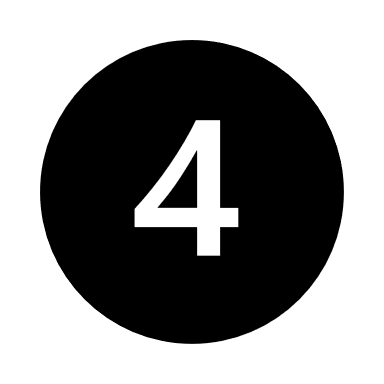 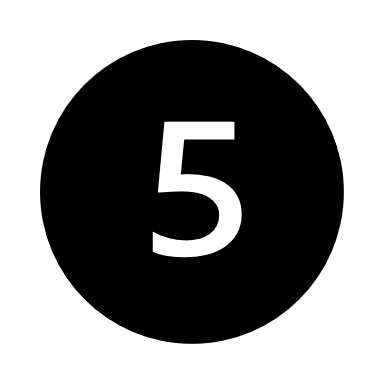  Extremely Extremely  Unimportant Important |
| During my assessment interview, I could understand what the assessor said to me  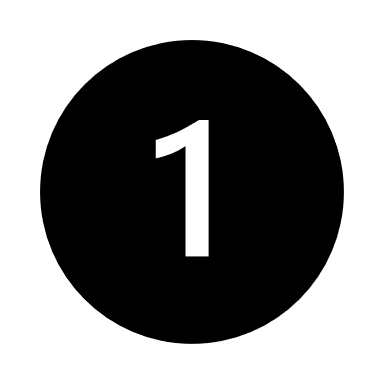  **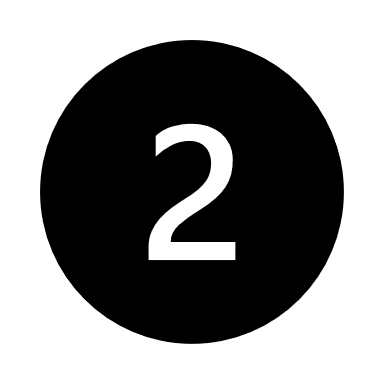** 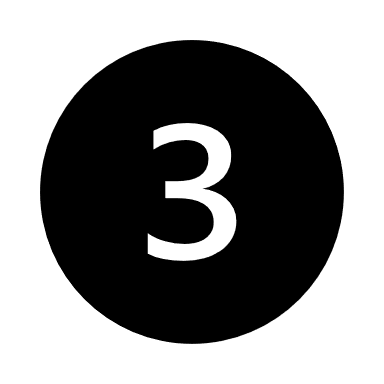 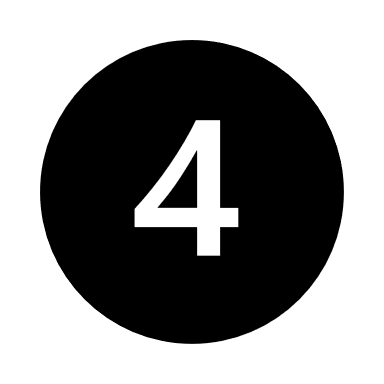 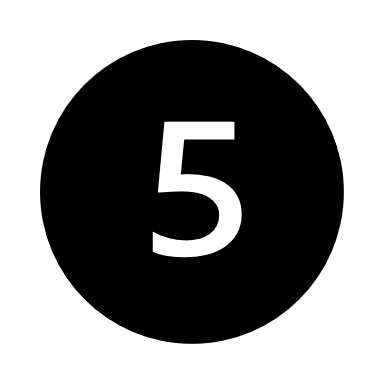  Extremely Extremely  Unimportant Important |
| During my assessment interview, my cultural and/or religious preferences were respected  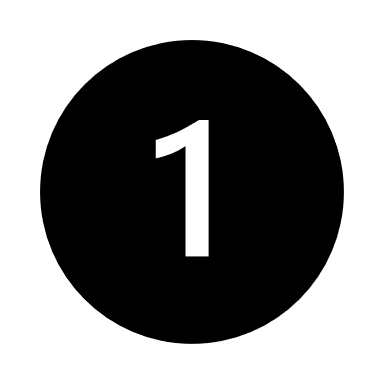  **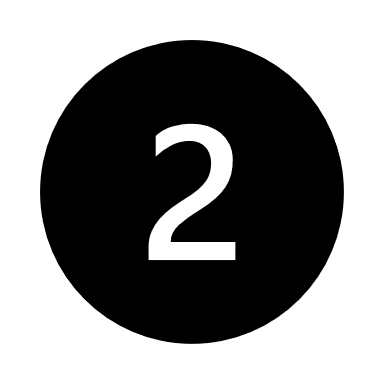** 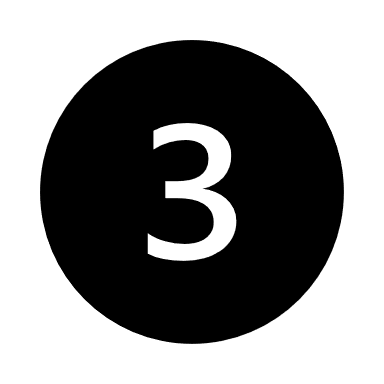 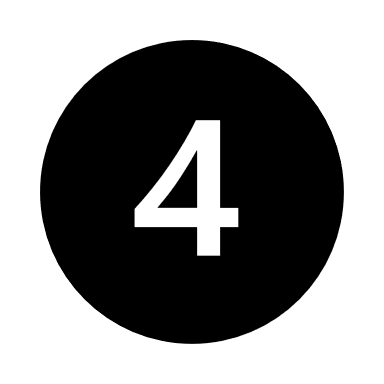 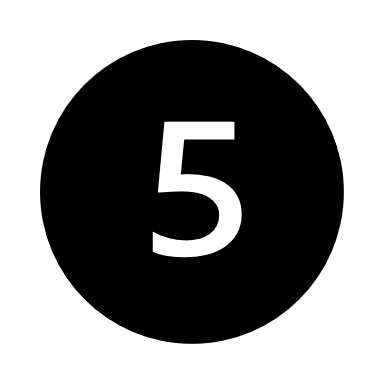  Extremely Extremely  Unimportant Important |
| During my assessment interview, I was treated with dignity and respect  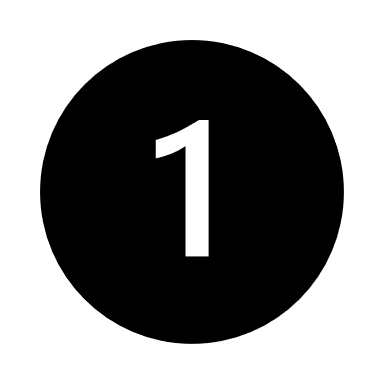  **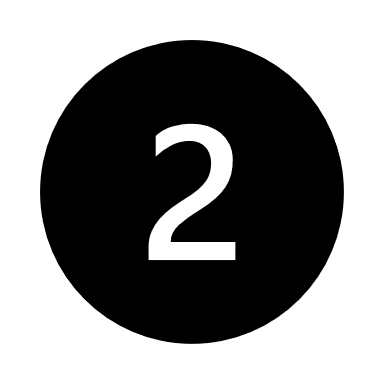** 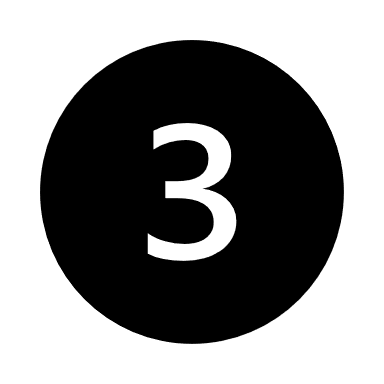 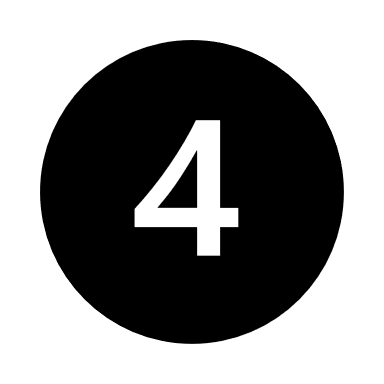 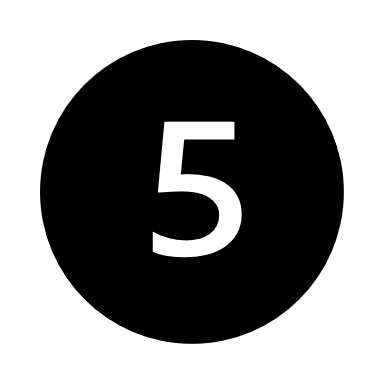  Extremely Extremely  Unimportant Important |
| During my assessment interview, I was supported to raise any concerns about getting the help I need  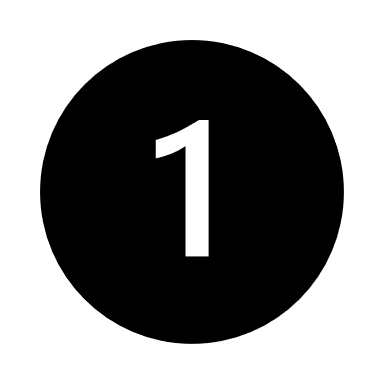  **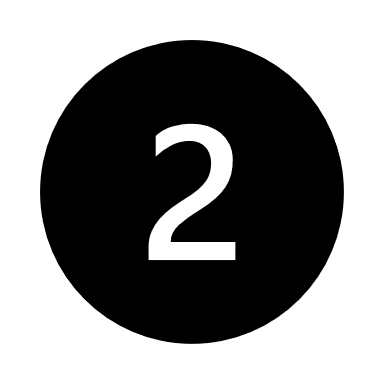** 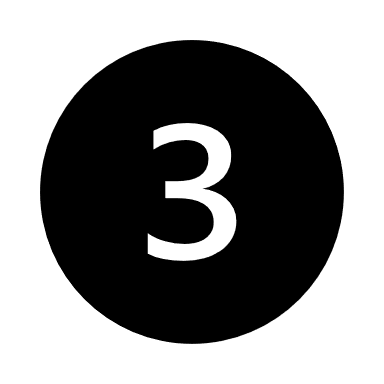 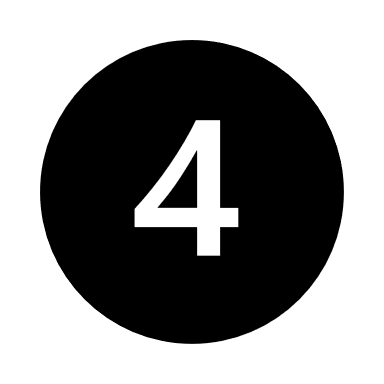 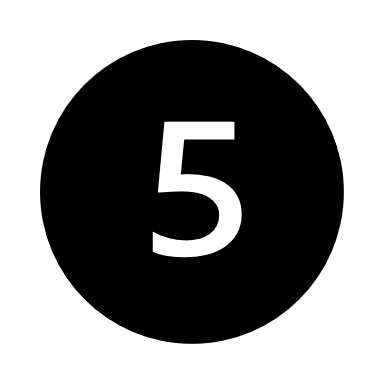  Extremely Extremely  Unimportant Important |
| During my assessment interview, I was treated as an equal partner in the care planning process  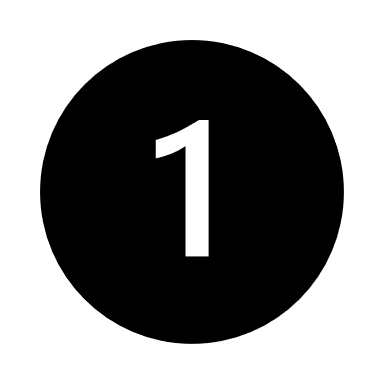  **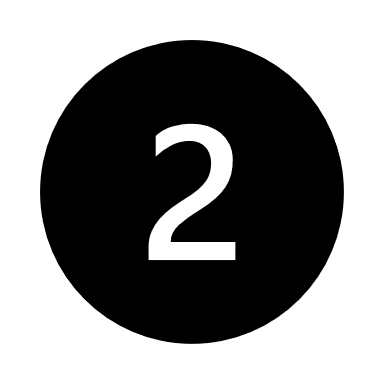** 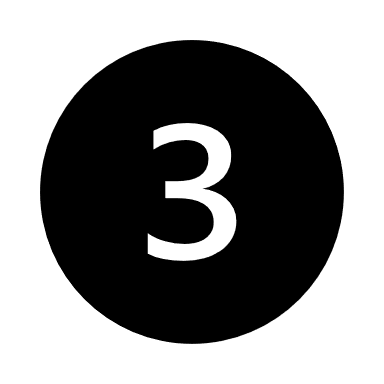 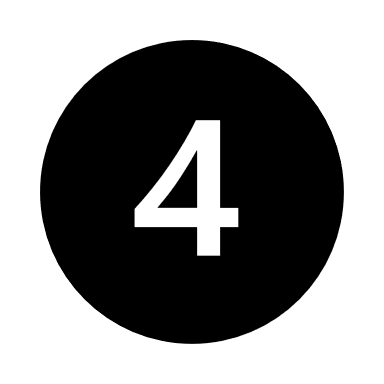 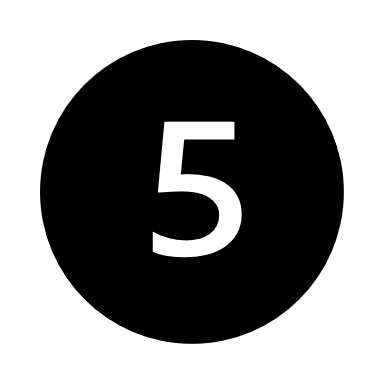  Extremely Extremely  Unimportant Important |
| During my assessment interview, I was provided with opportunities to make decision about my care needs  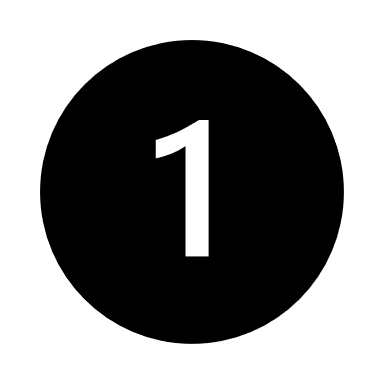  **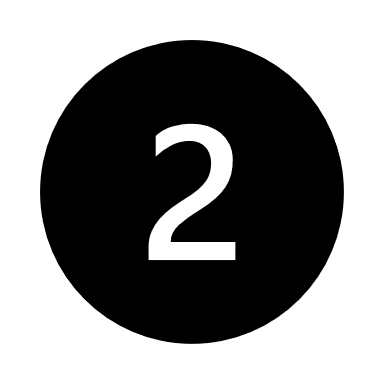** 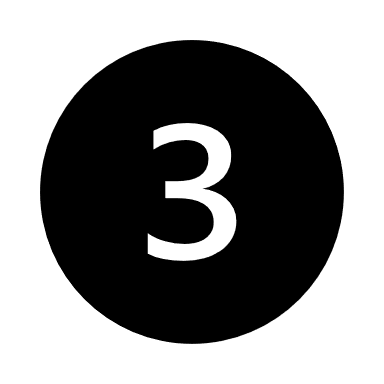 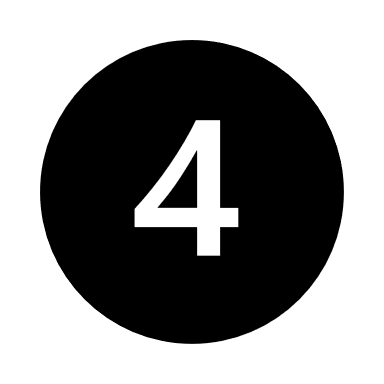 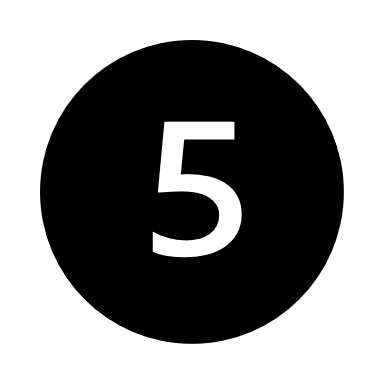  Extremely Extremely  Unimportant Important |
| During my assessment interview, I had enough time to talk with the assessor  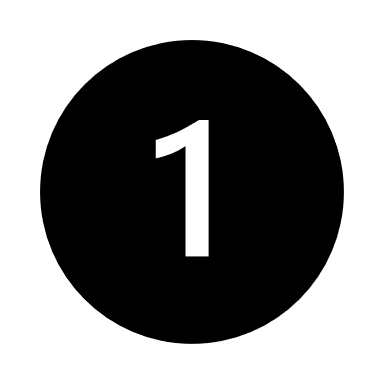  **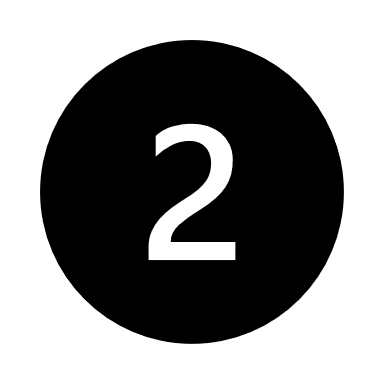** 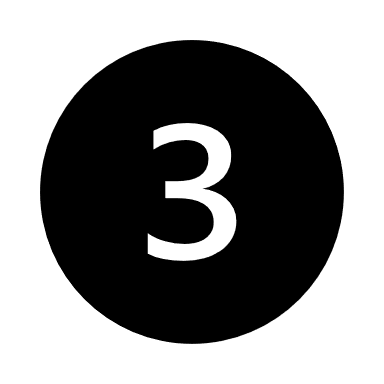 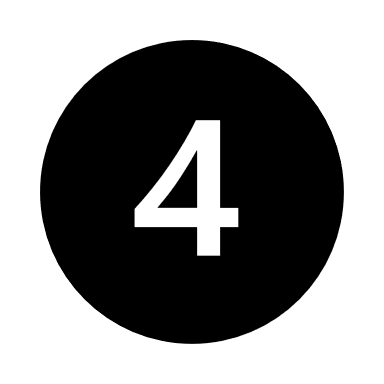 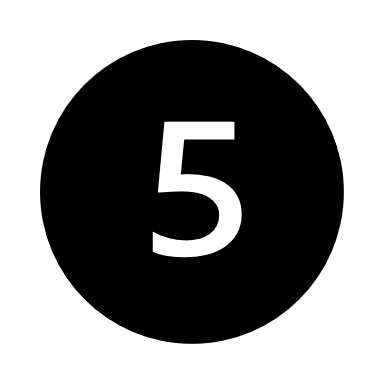  Extremely Extremely  Unimportant Important |
| During my assessment interview, I had enough time to make decisions  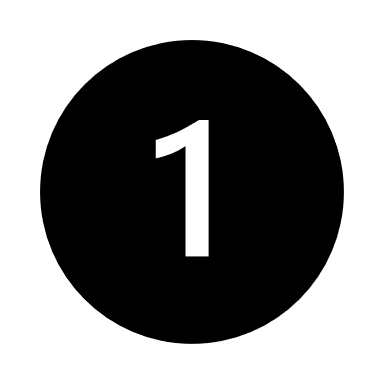  **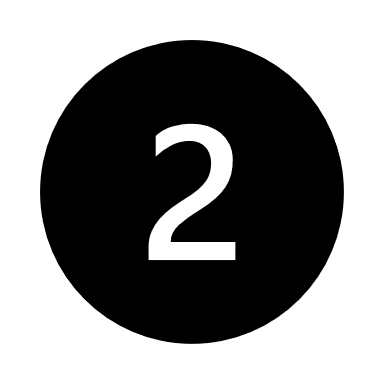** 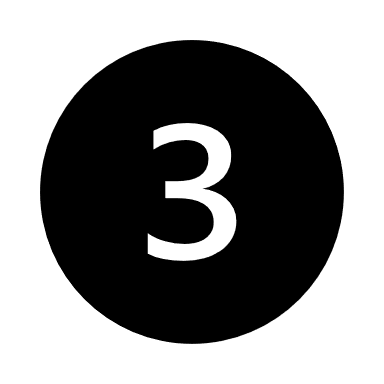 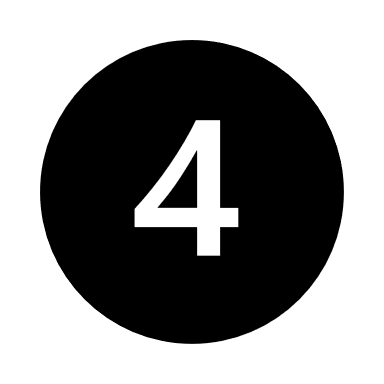 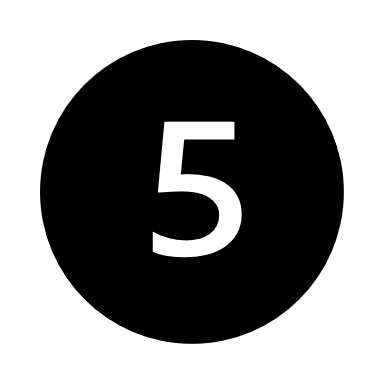  Extremely Extremely  Unimportant Important |
| During my assessment interview, the assessor explained the responsibilities of the Aged Care Assessment Team  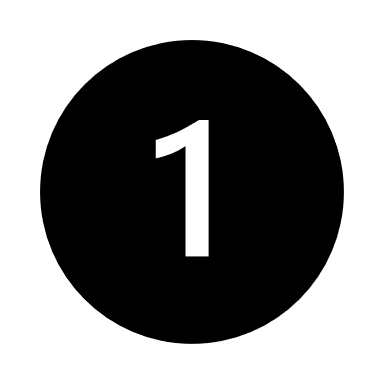  **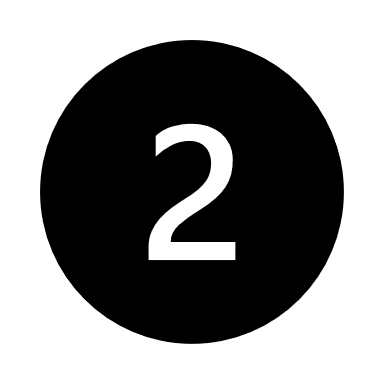** 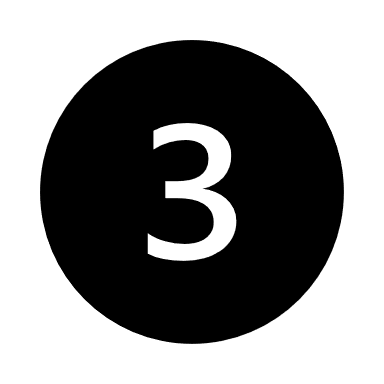 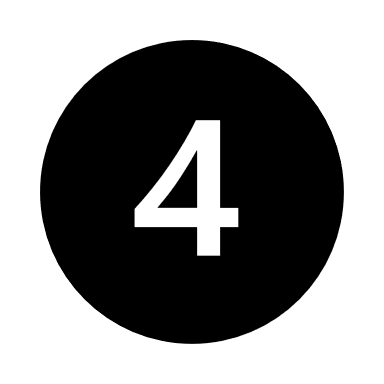 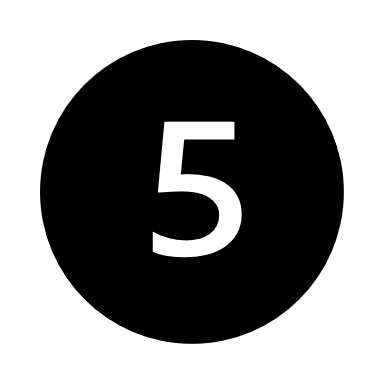  Extremely Extremely  Unimportant Important |
| After my assessment interview was completed, the assessor explained what the next steps were  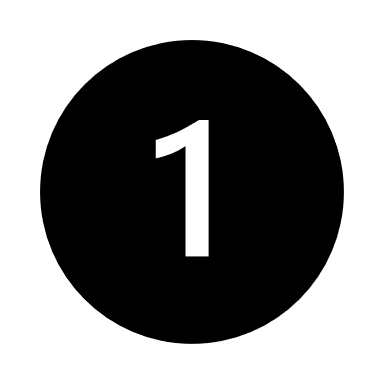  **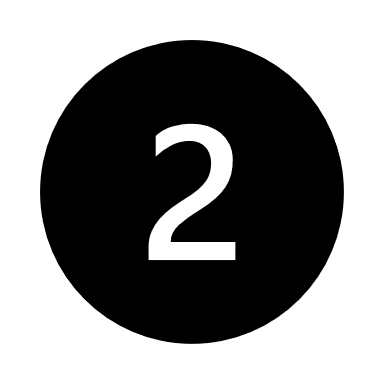** 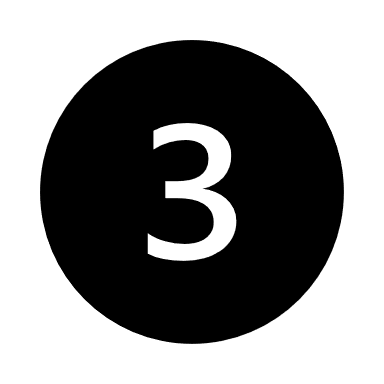 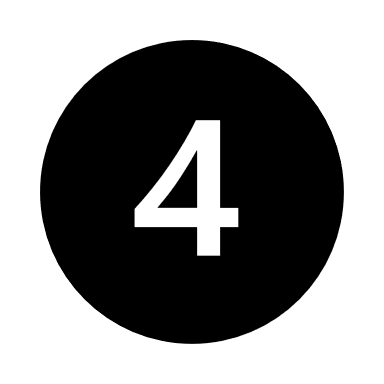 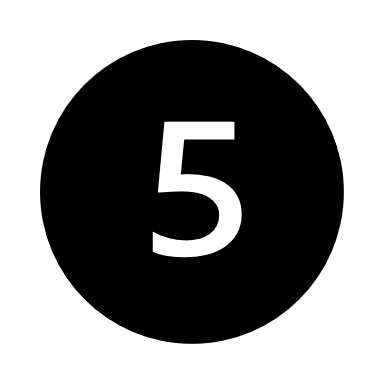  Extremely Extremely  Unimportant Important |
| After my assessment interview was completed, the assessor explained what I was expected to do next  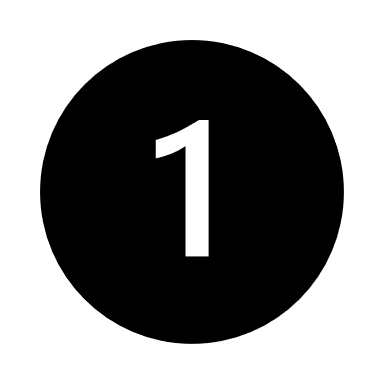  **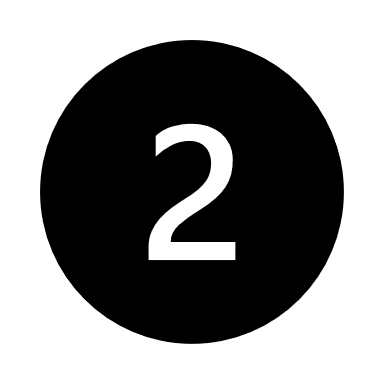** 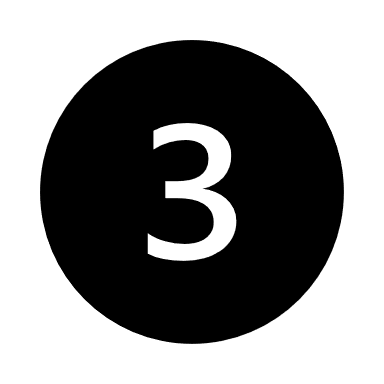 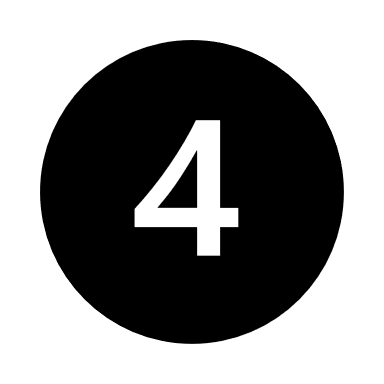  Extremely Extremely  Unimportant Important |
| Written information I received explaining my care needs reflected dignity and respect    Extremely Extremely  Unimportant Important |
| Written information I received acknowledged the importance of cultural and spiritual preferences    Extremely Extremely  Unimportant Important |
| The support plan summary I received reflected my needs    Extremely Extremely  Unimportant Important |
| If my care needs change after my assessment has been completed, I know who I can ask for assistance    Extremely Extremely  Unimportant Important |
| I know what care I am eligible to access    Extremely Extremely  Unimportant Important |
| The assessor explained that an Aged Care Assessment delegate would decide what Aged Care Assessment type I would be approved to access    Extremely Extremely  Unimportant Important |
| Information I was given at my assessment interview was easy to understand    Extremely Extremely  Unimportant Important |
